# Discovering Uncharted Binding Pockets on E3 Ligases Leads to the Identification of FBW7 Allosteric Modulators

**DOI:** 10.1002/advs.202506068

**Published:** 2025-08-20

**Authors:** Míriam Martínez‐Cartró, Álvaro Serrano‐Morrás, Andrea Bertran‐Mostazo, Roger Castaño‐Muñiz, Salvatore Scaffidi, Varbina Ivanova, Noémi Csorba, József Simon, Péter Ábrányi‐Balogh, Yunfeng Li, György M. Keserü, Bing Hao, Xavier Barril, Carles Galdeano

**Affiliations:** ^1^ Departament de Farmàcia i Tecnologia Farmacéutica i Fisicoquímica. Facultat de Farmàcia i Ciències de l'Alimentació Universitat de Barcelona Barcelona 08028 Spain; ^2^ Institut de Biomedicina de la Universitat de Barcelona (IBUB) Universitat de Barcelona Barcelona 08028 Spain; ^3^ Institut de Química Teòrica i Computacional (IQTC) Universitat de Barcelona Barcelona 08028 Spain; ^4^ Medicinal Chemistry Research Group. HUN‐REN Research Center for Natural Sciences Budapest 1117 Hungary; ^5^ National Drug Research and Development Laboratory. HUN‐REN Research Center for Natural Sciences Budapest 1117 Hungary; ^6^ Department of Organic Chemistry and Technology. Budapest University of Technology and Economics Budapest 1111 Hungary; ^7^ Department of Molecular Biology and Biophysics University of Connecticut School of Medicine Farmington CT 06030 USA; ^8^ Catalan Institution for Research and Advanced Studies (ICREA) Barcelona 08010 Spain

**Keywords:** Allosterism, drug discovery, E3 ligases, Ligandability/druggability, targeted protein degradation

## Abstract

E3 ligases are key regulators of the ubiquitin‐proteasome system (UPS) and have emerged as attractive drug target candidates for precise therapeutic intervention. Additionally, their ligands are extremely valuable as handles for Targeted Protein Degradation (TPD). However, only a limited number of E3 ligases have been targeted with small molecules. An efficient approach to identify ligandable surfaces on 22 structurally diverse E3 ligases has been developed, revealing that they offer significant binding opportunities through allosteric pockets. As a proof of concept, an allosteric pocket identified in FBW7 has been targeted, leading to the discovery of the first potent and reversible small‐molecule binders of this E3 ligase. Biophysical and structural studies have confirmed the binding site, while functional cell assays have showed that some of these molecules act as allosteric enhancers of c‐MYC and c‐JUN degradation in an FBW7‐dependent manner. These allosteric modulators of E3 ligases represent a novel mechanism of action in the TPD landscape and could be used as PROTAC handles.

## Graphical abstract

A computational approach to identify and characterize ligandable binding pockets on E3 ligases is presented. As a validation case, the first low‐micromolar small moleculesthat bind to the FBW7 E3 ligase are identified and confirmed to bind at the intended site. Some of the molecules identified are capable of allosterically enhance the natural degradation of c‐MYC and c‐JUN by FBW7 in an E3 ligase‐dependent manner.

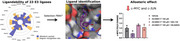

## 1 Introduction

E3 ligases are crucial components of the ubiquitin‐proteasome system (UPS) that confer substrate specificity for proteasomal degradation. E3 ligases have been linked to various pathogenic mechanisms, and for this reason, they have emerged as attractive drug target candidates for personalized medicine, eliciting more specific responses and fewer side effects than the proteasome inhibitors currently in use.^[^ ^1^ ^]^ Depending on the function of an E3 ligase, pharmacological modulation may seek to promote a higher rate of substrate degradation or, in contrast, inhibit its function. However, despite the tremendous clinical significance of this large protein family, only a handful of immunomodulatory drugs (IMiDs) have been approved, all of them targeting the cereblon (CRBN) E3 ligase.^[^ ^2^ ^]^

In parallel, the rise of targeted protein degradation (TPD) approaches has placed E3 ligases at the center of drug discovery efforts, as recruitment by an E3 ligase is a mandatory step for the mechanism of action of PROTACs and molecular glues.^[^ ^3^, ^4^ ^]^ Currently, only about a dozen E3 ligases have been exploited for TPD strategies, with von Hippel‐Lindau (VHL) and CRBN being the most hijacked.^[^ ^5^, ^6^ ^]^ Although both have consistently demonstrated their potential for degrading numerous targets via a “plug‐and‐play” approach, they still face chemical, biological, and toxicological limitations. The major challenge in hijacking E3 ligases beyond VHL and CRBN still lies in the lack of corresponding small‐molecule binders that could be incorporated into the degrader structure. Given that E3 ligases represent a large protein family with over 640 members, there is a clear mismatch between the number of family members and the number that have been successfully targeted by drug‐like small‐molecules.^[^ ^7^, ^8^ ^]^ This mismatch reflects, at least in part, the novelty and complexity of this protein family, but also indicates intrinsic difficulties in targeting them with small molecules (i.e., poor ligandability) and/or translating such molecules into effective drugs (i.e., poor druggability), which results in significant time and resources being spent in risky drug discovery programs.

Many of the recently identified E3 ligase‐targeting molecules have been discovered through phenotypic assays. This raises the question of whether these assays are a productive approach for expanding the E3 ligase toolbox or if they merely identify a small subset of E3 ligases, as discussed by Zhang et al.^[^ ^9^ ^]^ In contrast, “binder‐first” E3 ligase‐centered approaches may offer a more promising option for expanding the current set of targeted E3 ligases, regardless of their family function and/or structure. Such approaches could increase the structural diversity of targeted E3 ligases and binding pockets. Several members of the DCAF family have been successfully hijacked for TPD purposes with molecules containing highly reactive electrophilic groups.^[^ ^10^ ^]^ Nonetheless, these covalent molecules could raise concerns about specificity and drug development, as they may, for example, target cysteine residues located in unligandable regions of the E3 ligases, such as intrinsically disordered regions.^[^ ^11^ ^]^

Rational structure‐based approaches have been largely focused on targeting the degron recognition site of E3 ligases, a challenging and also risky endeavor, as molecules must compete with natural substrates.^[^ ^12^ ^]^ In parallel, fragment‐based campaigns have also met with considerable success, however, growing fragments to drug‐sized molecules can be very challenging.^[^ ^13^ ^]^ High‐throughput virtual screening (HTVS) has proven to be an effective tool for identifying novel chemical matter in drug discovery programs.^[^ ^14^ ^]^ This computational approach offers several advantages in terms of efficiency (time, accessibility, budget) compared to other “binder‐first” approaches such as DNA‐encoded library (DEL) and/or fragment screenings.^[^ ^12^, ^13^, ^15^, ^16^ ^]^ However, its full potential has not yet been realized for E3 ligases.

**Figure 1:**
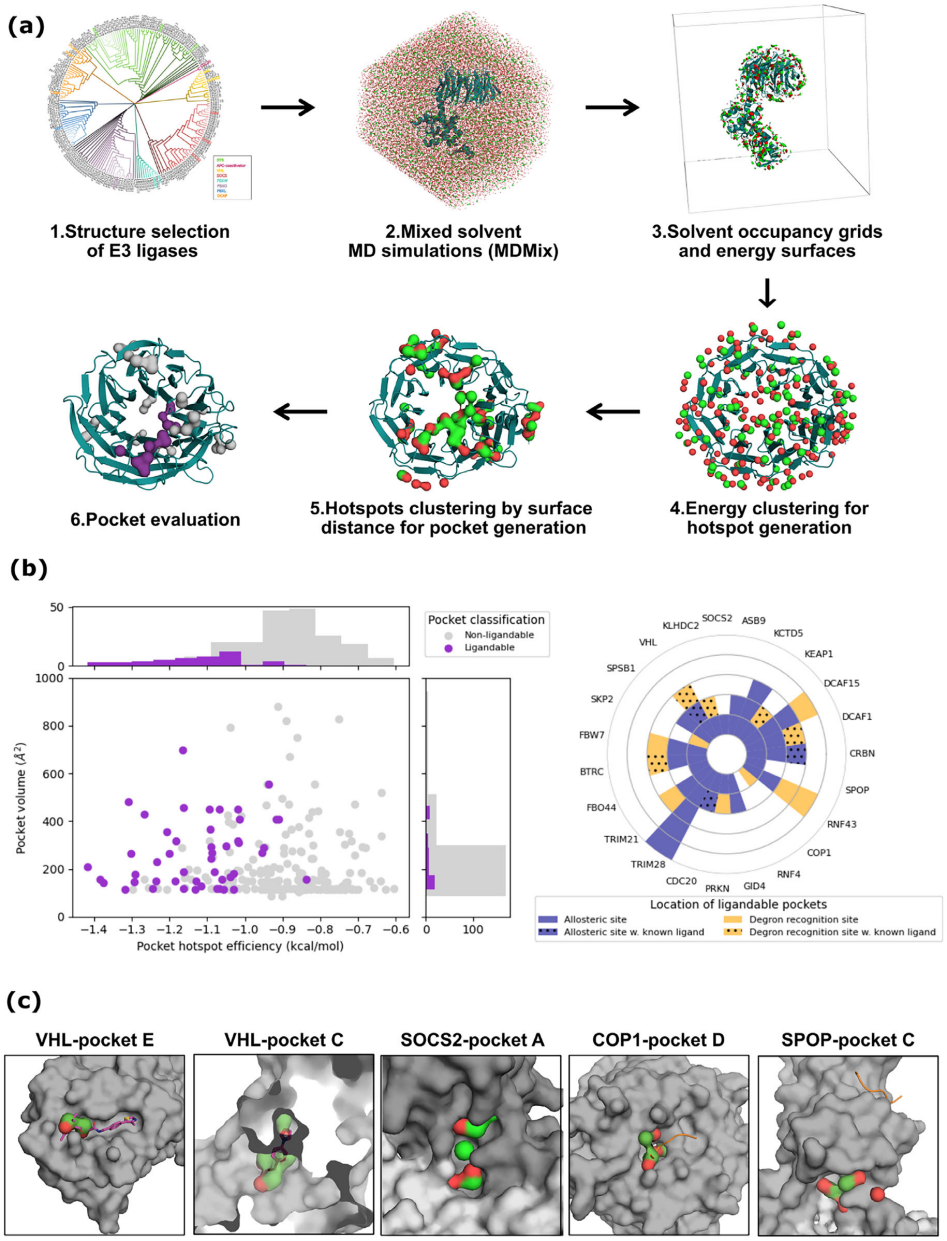
A validated computational approach to identify ligandable pockets on E3 ligases. a) Pipeline of the identification and evaluation of pockets through MDMix. The dendrogram was extracted from Ubihub and colored by structural families. Hydrophobic (green) and polar (red) densities and hotspots are shown of the ethanol solvent mixture. Ligandable pockets are highlighted in purple while the non‐ligandable in grey. b) Description of the identified pockets. The left plot shows the pockets’ hotspot efficiency (as sum of energies/no. of hotspots) to the pocket volume. On the right, the number of ligandable pockets identified per E3 ligase studied colored by their location (allosteric in blue and degron recognition sites in yellow) and presence of crystallized ligands reported (dotted pattern). c) Examples of ligandable pockets identified through the pipeline with their hotspots highlighted. From left to right VHL‐pocket E, VHL‐pocket C, SOCS2‐pocket A, COP1‐pocket D, and SPOP‐pocket C.

To expand current approaches and efficiently increase the arsenal of targeted E3 ligases, we present a validated and easy‐to‐implement computational method (**Figure** **1**a–c) (i) identify and characterize individual binding pockets on E3 ligases and (ii) enable the identification of small‐molecule that bind to them. As proof of concept, we reveal binding pockets on a diverse set of 23 E3 ligases, then proceed to identify the first small‐molecule ligands for the FBW7 E3 ligase by targeting an identified allosteric pocket. Biological studies with these allosteric molecules show that some of them can enhance the pharmacological degradation of c‐MYC and c‐JUN, natural substrates of the FBW7 E3 ligase,^[^ ^14^, ^17^ ^]^ in an E3 ligase‐dependent manner.

## 2 Results and Discussion

### 2.1 A Validated Approach to Identify Ligandable Pockets on E3 Ligases

We compiled a comprehensive database of all available E3 ligases with X‐ray structures, including both structural (e.g. crystal resolution, % sequence coverage) and functional (e.g. small‐molecules ligands, predicted relevance for protein degradation) data. Structural information was sourced from the PDB, while functional annotations were obtained from Ubihub,^[^ ^18^ ^]^ Uniprot and PROSITE. Using these data, we chose a representative set of 23 E3 ligases based on four comprehensive criteria: i) availability of high‐resolution X‐ray structures (<2.5 Å) with great coverage of the substrate‐recognition; ii) representation of diverse structural motifs within the substrate‐recognition domain; iii) well‐documented biological relevance and iv) presence or absence of reported small molecules known to directly bind to the E3 ligase. Both untargeted E3 ligases and E3 ligases with known small‐molecule ligands were included to allow for retrospective and prospective validation of our computational approach, respectively (Figures S1 and S2a,b, Supporting Information).

To assess ligandability and identify binding pockets, we used mixed‐solvent molecular dynamics simulations which allows the obtention of binding hot spots (with their corresponding binding free energy) for polar and hydrophobic atom types.^[^ ^19^, ^20^ ^]^ Adaptor proteins (e.g. SKP1, DDB1, elongins) were retained in these simulations to avoid introducing noise from inaccessible protein‐protein binding pockets. We clustered the most energetic points on the E3 ligases' surfaces using surface distance as the descriptor. To ensure the ligandability of these pockets, we set a minimum of four hotspots per cluster, all within a maximum volume of 500 Å^3^. The resulting binding pockets were further filtered based on their geometry and balancing polar and hydrophobic hotspots, a feature required for ligandability.^[^ ^21^ ^]^ Finally, we ranked these binding pockets by summing the energies derived from their hotspots. Overall, the finally selected binding pockets showed similar physicochemical characteristics but with higher energetic efficiency (i.e. overall energy of their interaction hotspots) (Figure 1b; Figure S3, Supporting Information).

To retrospectively validate the binding pocket predictions, we focused on E3 ligases that have been crystallized with non‐covalent small‐molecules. As an illustrative example, VHL degron recognition is driven by the substrates’ hydroxyproline, including its natural substrate HIF‐1α. Potent VHL molecules containing this chemical moiety have been described^[^ ^22^, ^23^ ^]^ and extensively used for the development of PROTAC molecules.^[^ ^24^, ^25^ ^]^ Remarkably, one of the identified pockets on VHL with favorable energy profiles (VHL‐pocket E, ‐6.3 kcal/mol) corresponds to the HIF‐1α degron site. The two main hotspots of this VHL‐pocket E match perfectly with the hydroxyl and *tert*‐butyl binding surfaces, characteristics of reported VHL inhibitors (Figure 1c). Importantly, the energetics obtained match very well with the *tert*‐butyl‐*trans*‐hydroxyproline molecule described (two‐digits micromolar).^[^ ^26^ ^]^ We also identified partially the IMiDs binding pocket in CRBN (CRBN‐pocket G, −5.6 kcal mol^−1^)^[^ ^27^, ^28^ ^]^ and additional targeted pockets in E3 ligases such as KEAP1 (KEAP1‐pocket B, −9.6 kcal mol^−1^)^[^ ^29^, ^30^ ^]^ and βTRC (βTRC‐pocket C, −6.4 kcal mol^−1^).^[^ ^31^ ^]^ Furthermore, we found as ligandable non‐targeted degron site pockets, such as COP1‐pocket D (‐9.3 kcal mol^−1^), which mirrors TRIB1 peptide interactions (Figure 1c). Due to the mild protein restraints used during the mixed‐solvent MD simulations and the hydrophobicity of ethanol, cryptic binding pockets and conformational changes were seen in some E3 ligases. As an example, in the case of VHL, we identified an additional binding site, VHL‐pocket C (9.6 kcal mol^−1^), which corresponds to a previously described allosteric binding site in a fragment‐based campaign (Figure 1c).^[^ ^32^ ^]^

A significant number (66%) of the identified binding pockets were novel (i.e., previously unreported) and allosteric (Figure 1b). As an example, while we encountered challenges reproducing the ligandability of degron binding sites in the SOCS‐BOX family due to their flat surfaces, we identified a highly energetic allosteric pocket in SOCS2 (SOCS2‐pocket A, −8.7 kcal mol^−1^), located near the interface with elongin C, which was opened during simulation. In a different structural context, we also predicted an energetic binding pocket in a highly interesting region of the SPOP E3 ligase. The SPOP‐Pocket C (−7.63 kcal mol^−1^) is in a hinge region, behind the degron recognition site, which could introduce functional effects if altered. (Figure 1c) In fact, allosteric pockets offer unique opportunities for TPD strategies, as their ligands do not compete with the natural substrates, requiring lower affinity and being less likely to interfere with the natural function of the E3 ligase. Furthermore, they offer increased selectivity and avoid resistant‐inducing mutations. The use of allosteric molecules can increase the number of targeted E3 ligases, advancing the development of tissue‐ and disease‐specific degraders.^[^ ^8^, ^33^ ^]^

**Figure 2:**
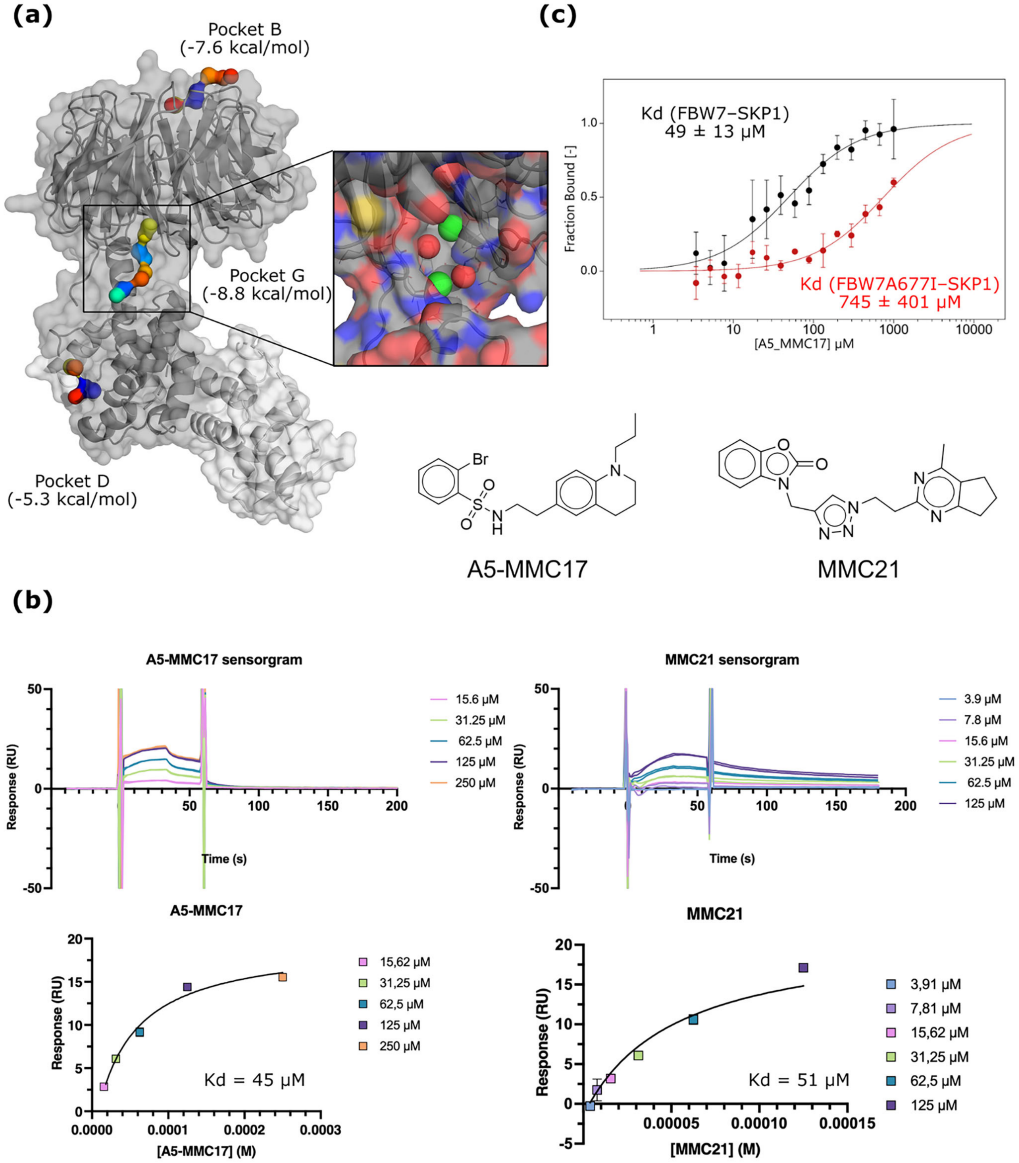
Identification of the first potent small‐molecules targeting the FBW7 E3 ligase. a) The three identified FBW7 pockets, colored by their associated energy values per hotspot. FBW7 (grey) and SKP1 (light grey) are represented with their secondary structure and their Van der Waals surface. FBW7‐pocket G is zoomed in, where the four pharmacophores are represented as spheres (hydrophobic in green and polar in red). b) Chemical structures and SPR results for two of the molecules tested (A5‐MMC17 and MMC21). Sensorgrams (above) and thermodynamic fitted curves (bottom) for both examples. c) MST results comparing the binding of A5‐MMC17 to FBW7^WT^‐SKP1 (black) and FBW7^A677I^‐SKP1 (red).

WD40 repeat domain (WDR) was the most represented structural motif in our selection of E3 ligases. For E3 ligases within this family, we identified potential binding pockets outside of the degron recognition sites i.e. allosteric in CDC20, βTRC, and FBW7. In particular, the FBW7 E3 ligase exhibited an interesting profile of pockets (**Figure** **2**a). FBW7‐pocket B (−7.6 kcal mol^−1^) is located on top of the WDR domain, near the degron recognition site. Additionally, we identified two pockets distal from the degron site with potential allosteric effects: FBW7‐pocket D (−5.3 kcal mol^−1^) and FBW7‐pocket G (−8.8 kcal mol^−1^). FBW7‐pocket D lies at the interface between the F‐box and SKP1, while FBW7‐pocket G is situated in the linker region between the WDR and F‐box domains. The flexibility between the WDR and linker domains has been described to play a crucial role in substrate ubiquitination in this family of E3 ligases.^[^ ^34^, ^35^ ^]^ WDR domains have been recognized as a good target not only in the E3 ligase family^[^ ^36^ ^]^ but efforts have been mainly focused on targeting surfaces in the central pocket and their ligandability classification is based on this site.^[^ ^37^ ^]^

Overall, our tool resulted in the identification of ligandable pockets in 22 out of 23 E3 ligases studied, with an average of 2.3 pockets per E3 ligase. The excellent agreement between the already known targeted binding pockets and those predicted, strongly supports the accuracy and reliability of our approach. It also highlights many yet‐unexplored pockets in E3 ligases suitable for designing novel and/or improved small‐molecules. Ligandability maps of E3 ligases have been already described using chemo‐proteomics approaches, however they are restricted to identifying pockets close to reactive cysteines.^[^ ^10^ ^]^ In parallel, many computational tools have been reported in the literature to predict ligandable pockets.^[^ ^38^, ^39^, ^40^, ^41^ ^]^ However, most represent receptors as static entities or cover only a limited range of conformational variability, including E3 ligases.^[^ ^42^ ^]^ Remarkably empirical druggability methods are trained on datasets that contain relatively few allosteric pockets.^[^ ^43^, ^44^ ^]^ As a result, it is unclear whether these methods can effectively detect such pockets. In contrast, physics‐based methods like the one employed here offer general applicability, through a higher computational cost.^[^ ^21^ ^]^ Specifically, molecular simulations with organic solvents have been reported to facilitate the opening of cryptic pockets and ligand‐induced conformational changes.^[^ ^45^, ^46^ ^]^

We chose to build a smaller selection of E3 ligases and propose a high level of curation of the results with careful conformational analysis. However, we restricted ourselves to X‐ray structures of E3 ligases, which limited the sequence coverage of our input structures and the availability of relevant functional domains and adaptor proteins. Nonetheless, adapting the workflow to use AlphaFold‐predicted structures and complexes should be straightforward and should not negatively impact the predictions. Futhermore, the computational approach described herein can be applied to a wide range of protein families. However, it is important to consider that the specific parameters for clustering energy hotspots to automatically assign pocket ligandability may vary depending on the desired nature of the ligands (e.g., small molecules, peptides, beyond rule‐of‐five compounds) and the pockets. Naturally, the approach remains limited to well‐structured proteins and is not applicable to protein regions with an abundance of disordered sequences.

### 2.2 Identification of the Potent Small‐Molecules Targeting the FBW7‐Pocket G

Considering that the FBW7 E3 ligase had not previously been targeted with potent small molecules, the allosteric nature of the pocket, the favorable energetic profile in terms of absolute ΔG_bind_ and pocket efficiency (Figure S3, Supporting Information), and the proposed relevance of the pocket in substrate ubiquination,^[^ ^34^, ^35^ ^]^ we selected FBW7‐pocket G as a test case to demonstrate the ability of our approach to identify allosteric small molecules. Furthermore, the high conservation of residues in FBW7‐pocket G across orthologous proteins also suggest a potential biological function for this pocket (Figure S4, Supporting Information).^[^ ^47^ ^]^ FBW7 is the substrate‐recognition member of the SKP1‐CUL1‐F‐box (SCF) degradation complex family. Its native substrates include key oncoproteins such as Cyclin‐E, c‐MYC, Notch, c‐JUN, mTOR, Mcl‐1, and AURKA.^[^ ^17^ ^]^ The degron pattern recognized by FBW7 is a double‐phosphorylation motif.^[^ ^48^ ^]^ FBW7 consists of three main domains: an *N*‐terminal F‐box domain that connects to the adaptor protein SKP1, a *C*‐terminal WDR domain that recruits substrates for degradation and a hinge/ flexible region that connects the two previous domains. FBW7 is one of the most deregulated proteins of the UPS in human cancers. Consistent with its role as a tumour suppressor, ≈6% of cancers present mutations in the *fbxw7* gene,^[^ ^49^ ^]^ with some leukaemia's (e.g. T‐ALL) showing a particularly high frequency of FBW7 mutations.^[^ ^50^ ^]^

To further validate the binding pockets identified using our computational approach and specifically FBW7‐pocket G, we applied the PhotoXplorer screening technology to the FBW7 E3 ligase.^[^ ^51^ ^]^ The PhotoXplorer library is composed by 100 representative fragments that cover most of the experimentally validated 2‐ and 3‐point fragment pharmacophores. The fragments are equipped with a photoaffinity handle that allows the identification of ligandable surfaces by mass spectrometry. Initial screening identified six hits, each showing an occupancy level above 5%. We selected the top 3 binders (Php060, Php006, and Php013, Figure S5, Supporting Information) for further evaluation and identified the exact site of labelling at residue level for these fragments using tryptic digestion followed by LC‐MS/MS measurements. The results suggest that the fragments were recognized by the target site before the binding mode was fixed by photoactivation. Residues Q306 and C308, labeled by Php006 and Php060, and T307 and R309, labeled by Php060, are positioned near FBW7‐pocket D (in the F‐box domain). Remarkably, residue C390, labeled by Php013, is located near FBW7‐pocket G (Figure S5, Supporting Information). To explore whether Php013 could occupy the selected FBW7‐pocket G, we performed molecular modelling on the FBW7‐Php013 covalent complex using docking and MD simulations. Starting from three different docking poses, we conducted unrestrained MD simulations and identified a stable mode of Php013 in FBW7‐pocket G (Figure S5, Supporting Information). The stability of the covalent complex was further confirmed with five additional free MD simulations. These results confirmed the ligandability of the binding pockets identified computationally, and in particular the ligandability of FBW7‐pocket G.

The interaction hotspots within FBW7‐pocket G were transformed into pharmacophoric restraints for HTVS. An in‐house curated virtual library of seven million molecules was screened using rDock,^[^ ^52^ ^]^ resulting in ≈0.5 million molecules with favorable docking scores. To reduce the number of molecules while preserving chemical diversity, a similarity clustering was performed, resulting in ca. 2,450 molecules. These were further filtered using the dynamic undocking (DUck) protocol,^[^ ^53^ ^]^ with a W_QB_ > 4 kcal·mol^−1^ threshold, leading to 62 potential molecules that passed all filters. Finally, 41 molecules were selected, purchased and tested for binding to FBW7 using Surface Plasmon Resonance (SPR) (Figure S6, Supporting Information). Nine of those exhibited a dose‐response sensorgram with steady‐state *K*_d_s in the one‐ to three‐digit micromolar range (Figure S7, Supporting Information), yielding a hit rate of 22% positive binders. Additionally, a structure‐activity relationship (SAR) by catalogue was performed for some of the 9 hits, purchasing 10 additional analogues that passed the docking‐based filters. These analogue molecules showed all *K*_d_s in the one‐ to three‐digit micromolar range, with a slight increase in potency compared to their parent molecules. Overall, these molecules represent the first potent reversible molecules targeting the FBW7 E3 ligase. Until now, only a non‐drug‐like biaryl molecule, originally discovered to bind reversible the β‐propeller blades of the ortholog CDC4 E3 ligase in yeast, has been previously reported to reversibly bind FBW7 with a *K*_d_ in the three‐digit micromolar range.^[^ ^54^ ^]^ Remarkably, MMC21 and MMC17 analogues (i.e. A4‐MMC17, A5‐MMC17, and A6‐MMC17) exhibited interesting slow association/dissociation profiles (Figure 2b).

Co‐crystallization and soaking experiments were performed for several of the parent molecules (MMC2, MMC17, and MMC21) with FBW7. Four datasets were collected for MMC2 and MMC17 crystals: one from MMC2 co‐crystallized with FBW7‐SKP1, and three from FBW7‐SKP1 crystals soaked with MMC17 (two datasets) and MMC2 (one dataset). Unfortunately, no additional electron density corresponding to the molecules identified was found in the FBW7‐pocket G in the determined structures. In the case of the soaking experiments, this can be due to a closed configuration of the pocket after the initial crystal formation. However, in all crystals, the electron density consistent with a sulphate ion from the crystallization solution (1.2 m Li_2_SO_4_ was used) was observed (Figure S10, Supporting Information). This density indirectly confirms that FBW7‐pocket G is a hotspot capable of binding small‐molecules. Moreover, when compared to the apo FBW7‐SKP1 published structure (PDB code 2OVP),^[^ ^48^ ^]^ the binding site pocket appeared slightly open, like the conformation of FBW7‐pocket G identified computationally. A particular challenge in the crystallization experiments was that FBW7‐SKP1 crystals do not tolerate DMSO, which complicated the solubilization of compounds at the required high concentrations. Considering this, an alternative approach to obtain information about the specific binding mode of these molecules could be cryo‐EM. This technique is more resistant to variations in buffer composition, such as the addition of DMSO required for molecule solubilization. However, the flexibility of the FBW7‐pocket G also presents a challenge for this technique.

To validate that the identified molecules target FBW7‐pocket G, the binding of selected molecules was compared in the wild‐type (WT) and a mutated form of FBW7 using orthogonal biophysical techniques. Site‐directed mutagenesis was performed on A677 of FBW7‐pocket G; introducing a bulkier amino acid (Ile) that is expected to interfere with ligand binding but, according to MutateX, should not affect protein stability (Figure S8, Supporting Information).^[^ ^55^, ^56^ ^]^ Affinity for FBW7^WT^ and FBW7^A677I^ was determined first for three of the best molecules (A5‐MMC17, A6‐MMC17, and MMC21) by microscale thermophoresis (MST). Each molecule was screened at different concentrations depending on its solubility and previous observed *K*_d_. The *K*_d_ obtained for FBW7^WT^ was in the same range as the initial SPR experiments. Differences in affinity between FBW7^WT^ and FBW7^A677I^ were observed for both A5‐MMC17 (Figure 2c) and A6‐MMC17. Surprisingly, no significant differences in affinity were observed for MMC21, suggesting that this molecule may not bind to FBW7‐pocket G. In all cases, changes in the initial fluorescence were observed, indicating that binding affinity should be assessed from these values rather than the MST signal. An SD‐test was performed to confirm that the fluorescence changes were caused by molecule interaction with the protein and no by artifacts. To further validate the observed differences between FBW7^WT^ and FBW7^A677I^, Isothermal Titration Calorimetry (ITC) and SPR experiments were conducted with A5‐MMC17 against both proteins (Figure S9, Supporting Information). In ITC, its binding to FBW7^WT^ showed a *K*_d_ of 51 µm and a ΔG of −5.85 kcal mol^−1^, consistent with initial SPR and MST experiments. In contrast, no measurable binding was observed when A5‐MMC17 was titrated with FBW7^A677I^. Similarly, SPR experiments also showed decreased binding of A5‐MMC17 for the mutated protein.

### 2.3 Allosteric Modulation of FBW7 Enhances c‐MYC and c‐JUN Degradation

**Figure 3:**
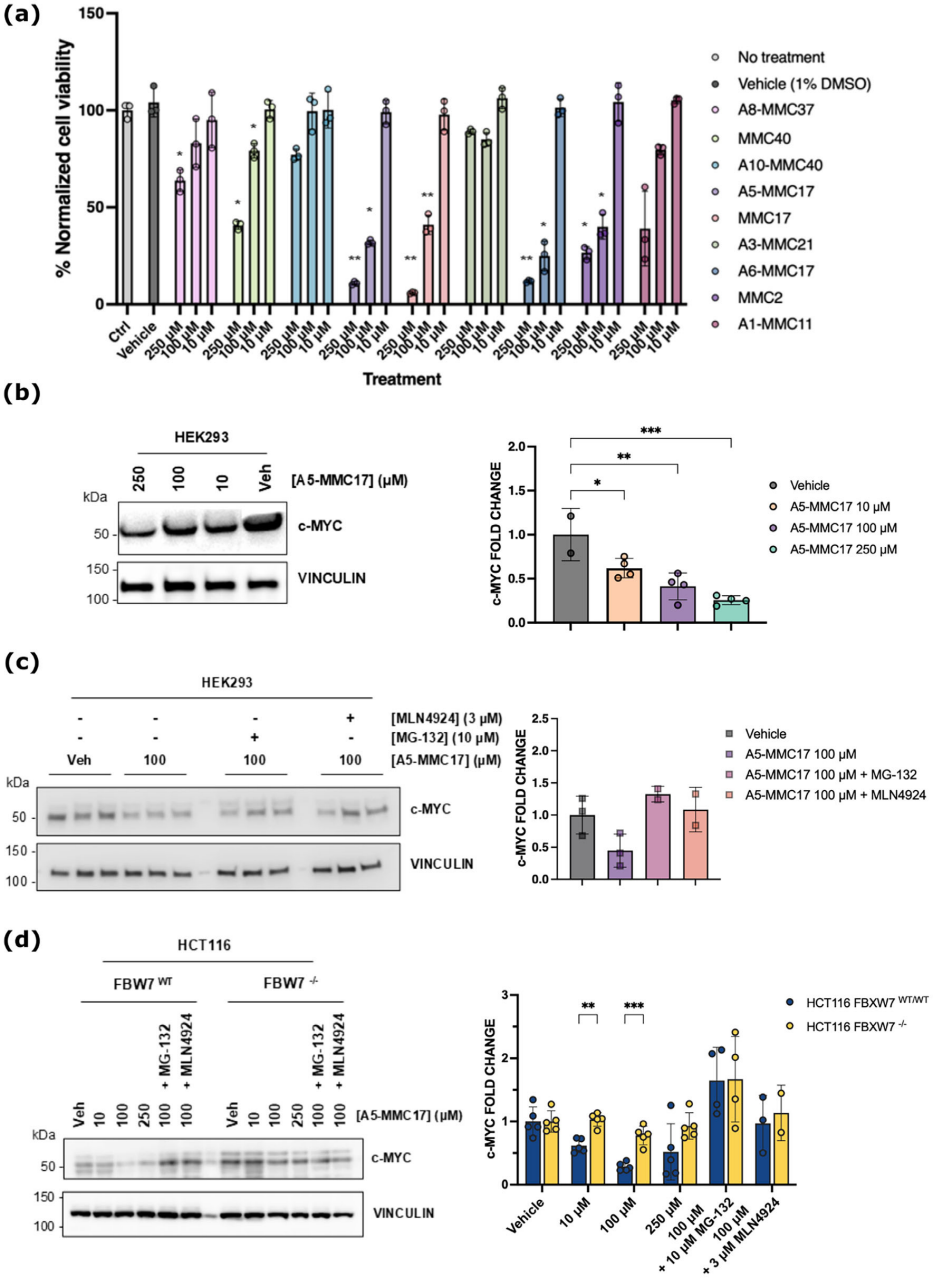
Allosteric modulation of FBW7 enhances c‐MYC and c‐JUN degradation. a) Evaluation of the cell viability after 2h of compound treatment. Results show three independent replicates for each condition (*n*=3). b) Immunoblotting results showing a dose‐dependent reduction of c‐MYC after 2h of A5‐MMC17 treatment in HEK293. Bar graphics illustrate the independent replicates showing the same result in HEK293 cells. c) Immunoblotting results after co‐treatment with MG‐132 and MLN4924. d) Immunoblotting results showing the on‐target effect resulting in a dose‐dependent c‐MYC degradation. Results show a clear reduction of c‐MYC after A5‐MMC17 treatment in FBW7^WT^ cells (blue), whereas it has a silent effect in FBW7^‐/‐^ cells (yellow). At least three independent replicates for each condition (*n*=3).

To assess whether the identified molecules trigger a possible functional effect through binding to the FBW7‐pocket G, a preliminary cell viability experiment was performed in HEK293 cells with some of the positive binders resulting from the SPR experiments. Very interestingly, several molecules significantly reduced the cell viability in a dose‐response manner (Figure 3a). Specifically, MMC17 and its analogues, A5‐MMC17 and A6‐MMC17, showed the most pronounced effects, reducing cell viability by ≈60%–70% at 100 µm, a concentration in the same range than the *K*_d_ obtained. Based on the previous biophysical studies and these phenotypic results, A5‐MMC17 was selected for further biological characterization.

To investigate the potential cause of the observed reduction in cell viability caused by some of the molecules, we evaluated the effect of A5‐MMC17 on the stability of FBW7 substrates. First, levels of c‐MYC in HEK293 and HCT116 cells following A5‐MMC17 treatment were measured. After 2 and 4 h of treatment, A5‐MMC17 caused a consistent dose‐dependent reduction in c‐MYC protein levels (Figure 3b). To confirm that the reduction in c‐MYC was not due to off‐target effects or toxicity, cells were co‐treated, 1h before compound treatment, with known inhibitors of the UPS pathway. Co‐treatment with MG‐132, a proteosome inhibitor, and A5‐MMC17 rescued c‐MYC levels, suggesting that the reduction in c‐MYC was due to proteasomal degradation (Figure 3c). Additionally, co‐treatment with MLN4924, a NEDD8 inhibitor, for 1 h before A5‐MMC17 treatment also counteracted the effect of A5‐MMC17 (Figure 3c). This demonstrates that the reduction in c‐MYC protein levels is also Cullin‐RING‐dependent. As a control, A8‐MMC37 (*K*_d_ = 23 µm), which did not affect cell viability, was not able to induce c‐MYC degradation (Figure S11, Supporting Information).

To further confirm that the reduction in c‐MYC protein levels was caused by the specific interaction of A5‐MMC17 with the FBW7 E3 ligase, the effect of A5‐MMC17 in the HCT116 (FBW7^WT^) cell line and a FBW7‐knockout (FBW7^‐/‐^) derivative of the parental HCT116 line was compared. Remarkably, in the HCT116 FBW7^‐/‐^ cells, A5‐MMC17 treatment did not affect c‐MYC protein levels at any concentration assayed. In contrast, in the HCT116 FBW7^WT^ cells, we observed a dose‐dependent, proteasome‐ and Cullin‐RING‐dependent degradation of c‐MYC, confirming the results obtained in HEK293 cells (Figure 3d).

Finally, to assess whether the allosteric degradation induced by A5‐MMC17 also occurs for other FBW7 substrates, cyclin E, MCL‐1, NOTCH, and c‐JUN were immunoblotted. While no degradation of cyclin E, MCL‐1, or NOTCH was observed (Figure S12, Supporting Information), a similar degradation pattern to the one observed for c‐MYC was seen for c‐JUN. Furthermore, this effect was not again observed in the HCT116 FBW7^‐/‐^ cells or when HCT116 FBW7^WT^ were treated with A8‐MMC37, demonstrating again the on‐target effect of A5‐MMC17 through binding to the identified FBW7‐pocket G. In comparison with other FBW7 substrates, c‐MYC and c‐JUN share some structural similarities, including a basic helix–loop–helix zipper (*b*HLHZip) region. This could suggest that these molecules might behave similarly when recruited by FBW7, which could potentially explain why the effect caused by A5‐MMC17 is only observed for these substrates of FBW7. Although the enhanced degradation of c‐MYC and c‐JUN was observed at micromolar concentrations due to the limited affinity of the molecules, on‐target effects were demonstrated by testing the molecules in an FBW7‐knockout cell line, suggesting a novel mechanism of action in TPD through positive allosteric modulation of an E3 ligase.

## 3 Conclusion

Here, we present an efficient and easy‐to‐implement approach based on inexpensive molecular dynamics simulations, to identify and characterize ligandable binding pockets on E3 ligases. All the ligandable pockets identified in this work are made publicly available, and we envision that this will spur the development of small molecules targeting E3 ligases. As a validation case, we conduct HTVS against a promising allosteric pocket of FBW7 using the novel dock‐DUck combination. We identify the first low‐micromolar reversible small‐molecules that bind to this E3 ligase and confirm that they bind at the intended site. Given their binding affinity, some of these molecules, in particular the ones that do not show allosteric modulation of FBW7, can be employed in the development of FBW7‐based PROTACs, an E3 ligase that has already proven its degradative capacity with chimeric and covalent approaches.^[^ ^57^, ^58^, ^59^ ^]^

Some of the molecules identified are capable of allosterically enhancing the natural degradation of c‐MYC and c‐JUN by FBW7 in an E3 ligase‐dependent manner. These results demonstrate the potential of “binder‐first” structure‐based approaches to exploit the druggability of E3 ligases, uncovering unsuspected allosteric functional binding sites and creating novel opportunities for pharmacological modulation of this protein family. These findings should inspire the development of similar programs in the field by targeting allosteric pockets on E3 ligases. Indeed, we encourage the scientific community to use all the ligandable pockets characterized in this work, which are openly shared, to facilitate the discovery of small molecules targeting new E3 ligases.

## 4 Experimental Section

### Compilation of E3 Ligases Dataset

A comprehensive database of all available E3 ligases with described structure was built, including structural and functional data. Ubihub^[^ ^18^ ^]^ was used to obtain the full list of E3 ligases and classify them based on their involvement in protein degradation (UPS score). Data from these resulting E3 ligases was downloaded from Protein Data Bank (PDB) including the resolution, sequence coverage, ligands. Structural and functional annotations were obtained from Uniprot and PROSITE. A representative structure was selected for each E3 ligase considering as selection criteria the best resolution, the sequence coverage and the presence of the degron recognition domain.

### Mixed‐Solvent Molecular Dynamics Simulations

The structures of the selected 23 E3 ligases are shown in Figure 2 (Supporting Information). Preparation of the structures (i.e. removal of crystallography artefacts, missing side chains, side chain protonation, loops reconstruction and capping) was performed with MOE 2016.^[^ ^60^ ^]^ The adaptor proteins present in ASB9, CRBN, DCAF15, FBXO44, FBXW7, SKP2, SOCS2, and VHL crystal structures were maintained.

Three 50 ns‐long replicas of mixed‐solvent molecular dynamics simulations of ethanol‐water (1:4) mixture were carried out for each E3 ligase. Simulations were performed using an octahedral solvation box with TIP3P waters, at 300K and 1 atmosphere. Protein heavy atoms were softly restrained (0.01 kcal·mol^−1^·Å^−2^). The solvation box for TRIM28 RBCC domain was changed to a prism to optimize the analysis. Simulations were performed using the ff14SB AMBER force field. For CRBN, TRIM28 (PHD‐BRD and RBCC domains), PRKN, and RNF4, zinc ions were parameterized using MCPB^[^ ^61^ ^]^ at the semiempirical level of theory.

Following the standard MDMix analysis,^[^ ^20^ ^]^ ethanol energy interaction patterns were extracted from its occupancy during the MD, separating the contributions in hydrophobic (corresponding to the alkyl tail) and polar (corresponding to the hydroxyl group). The highest energetic regions in the grids (top 0.02%) were clustered into interaction hotspots using the Boltzmann average. These hotspots were clustered using a procedure reported previously.^[^ ^62^ ^]^ The surface distance matrix was calculated between each hotspot considering the Van der Waals radii from protein atoms using the A* algorithm. Then the hotspots within 6Å were grouped in graphs with a minimum hotspot density of four. Second a graph merge step, gaining up to a 500 Å^3^ volume, was performed. When the MD simulations yielded high conformational variability, multiple conformations were used for the surface matrices. A final filtering step was performed ensuring good pocket geometry and a balance between polar and hydrophobic hotspots.

### PhotoXplorer Library Screening by Intact MS

The FBW7 protein was diluted to 200 µg mL^−1^ using pH 7.4 PBS buffer. To a clear plate 0.15 µL of 40 mm fragment solution in DMSO was added in each well and diluted with the protein solution to 15 µL. The protein/compound ratio was 1:100 and the final sample contained 1% DMSO. The plate was incubated at room temperature in the dark for 60 min, then cooled to 4 °C and irradiated at 365 nm for 10 min. The samples were then transferred into microvials and analyzed by LC‐MS‐TOF mass spectrometry (Agilent 1100 series liquid chromatography with Phenomenex SecurityGuard Widepore C4 4x3 mm cartridge with Sciex x500b QTOF system with electrospray ionization, positive mode, Data S1, Supporting Information). The deconvoluted spectra was analyzed using Sciex OS 2.0. software (version 2.0, Sciex).

### Digestion of the PhotoXplorer Hit Fragments – Sample Preparation

The protein was diluted to 1 mg/mL using pH 7.4 PBS buffer. 1 µL of 100 mm fragment solution in DMSO was added in each well and diluted with the protein solution to 50 µL (protein/compound ratio was 1:100 and the final sample contained 2% DMSO). The plate was incubated at RT in the dark for 60 min, then cooled to 4 °C and irradiated at 365 nm for 10 min. After the labelling was completed, 25 µL of the sample and 5 µL 0.2% (w/v) RapiGest SF (Waters, Milford, USA) solution buffered with 50 mm ammonium bicarbonate were mixed (pH 7.8) and 3 µL of 45 mm dithiotreitol (DTT) in 100 mm NH_4_HCO_3_ were added and kept at 37.5 °C for 30 min. After cooling the sample to room temperature, 3.5 µL of 100 mm iodoacetamide in 100 mm NH_4_HCO_3_ were added and placed in the dark at room temperature for 30 min. The reduced and alkylated protein was then digested by 3.5 µL (1 mg mL^−1^) trypsin (the enzyme‐to‐protein ratio was 1:10) (Sigma, St Louis, MO, USA). The sample was incubated at 37 °C for overnight. To degrade the surfactant, 3 µL of formic acid (500 mm) solution was added to the digested protein sample to obtain the final 40 mm concentration (pH 2) and was incubated at 37 °C for 45 min. For LC‐MS analysis, the acid treated sample was centrifuged for 5 min at 13 000 rpm and the supernatant was pipetted into a microvial. Samples were analyzed by a Triple TOF 5600+ hybrid Quadrupole‐TOF LC‐MS/MS system (Sciex, MA, USA) equipped with a DuoSpray IonSource coupled with a Shimadzu Prominence LC20 UFLC (Shimadzu, Japan). PeakView® V.2.2 software (version 2.2, Sciex) and Biologics Explorer software (version 750 3.0.3, Sciex) were used to assign and evaluate the MSMS spectra (Data S2, Supporting Information).

### Modelling FBW7‐PhotoXplorer Hit Complexes

The FBW7‐Php013 complex was built using a previously described tethered and constrained docking with rDock.^[^ ^52^ ^]^ The fragment core of the Phabit hit was positioned using one of the pharmacophores identified by MDMix while the diazirine warhead was tethered close to the labeled residue. The three highest scoring docking poses (based on SCORE.TOTAL) were used to assemble the covalently attached complex. MOE was used to link the protein residue with the warhead and perform initial minimization of the complex. The Php013‐labeled residue was parameterized using Gaussian and AmberTools.^[^ ^63^, ^64^ ^]^ Afterward, 500 ns of unconstrained MD simulations from the three starting positions of Php013 were performed. We clustered the trajectories to extract the representative structure of the most populated and stable Php013 binding mode. This binding mode was validated using 5 additional replicas of 100ns of classical MD (Figure S5, Supporting Information).

### Docking‐Based Virtual Screening

Docking‐based virtual screening with pharmacophoric restraints was performed with rDock.^[^ ^52^ ^]^ The cavity used for docking was defined in the prepared structure considering 8 Å radius from the MDMix hotspots of FBW7‐pocket G. An in‐house collection of 6 million molecules were docked to FBW7–SKP1 structure using three pharmacophoric restraints extracted from the MDMix hotspots (Figure S6, Supporting Information) using a tolerance of 0.5 Å for the polar pharmacophores and 1 Å for the hydrophobic ones. A HTVS filter was employed to optimize the docking calculations, with thresholds on SCORE.INTER (>‐18 to continue) and SCORE.RESTR.PHARMA (>1 to continue) In each stage the molecules were filtered depending on the “SCORE.INTER” (general docking score) and the “SCORE.RESTR.PHARMA” (pharmacophoric restraint score), continuing up to 50 iterations if the thresholds were fulfilled. These molecules were then clustered twice, first using the Reynold's algorithm and then the Jarvis‐Patrick in MOE, using an 85% similarity threshold and selecting the molecule with best SCORE.INTER from each cluster as representative. An additional docking run was executed with an extra polar pharmacophoric restraint (Figure S6, Supporting Information) but maintaining the other conditions.

### Applying Dynamic Undocking to Filter Docking Hits

Molecules from both HTVS campaigns were filtered with DUck,^[^ ^53^ ^]^ using the H‐bond with the N635 as a collective variable (CV). Molecules resulting from the second docking were also filtered using the H‐bond with S678 as an additional CV. Following DUck's standard procedure, the receptor was reduced to a “chunk”, maintaining the ligands’ environment with the residues within 9 Å of the ligand. ff99SB and pfrosst were used to obtain the protein and ligand parameters respectively. Ligand charges were derived using the AM1‐BCC model. After minimization and equilibration, steered molecular dynamics simulations (SMD) were performed at two different temperatures (300 and 325K), pulling the CV from 2.5 to 5.0 Å), with 0.5 ns sampling steps in between SMD. W_QB_ values (work necessary to break the hydrogen bond) were used to stop the simulation if lower than 4 kcal mol^−1^. The simulations were performed with GPU implementation of pmemd in AMBER20.^[^ ^65^ ^]^ These calculations were performed using the computational resources at the Barcelona Supercomputing Centre (BSC, Barcelona).

### Site‐Directed Mutagenesis of FBW7–SKP1 Mutants

To perform site‐directed mutagenesis of FBW7–SKP1, QuickChange II site‐directed mutagenesis kit (Agilent) was employed following the manufacturer's protocol. Primers’ design was performed following QuickChange manual guidelines and purchased in biomers.net. The complementary primers used to introduce the A677I point mutation were 5'‐CACCAGCTTTGTGTTTGAGATTCTGATCCGCCACACAACT‐3' and 5'‐AGTTGTGTGGCGGATCAGAATCTCAAACACAAAGCTGGTG‐3'. The polymerase chain reactions (PCR) were performed on 9700 GeneAmp® PCR System (Applied Biosystems®). Methylated and hemimethylated DNA was digested with DpnI for 1h at 37°C. The resulting product was transformed to XL1‐Blue competent cells. Amplified plasmid DNA was purified using GeneJet plasmid miniprep kit (Fisher Scientific) and sequenced by the Genomics Service of the *Centres Científics i Tecnològics de la Universitat de Barcelona* (CCiTUB) to confirm the presence of mutations.

### Protein Expression and Purification of FBW7–SKP1 and FBW7^A677I^–SKP1

The bicistronic plasmid encoding FBW7–SKP1 construct, with *N*‐terminal glutathione *S*‐transferase (GST)‐tagged human FBW7 (residue 263‐707) and truncated SKP1^[^ ^66^ ^]^ was cloned in a pAbloMut vector. The plasmid also contains a thrombin protease cleavage site after the tag and confers ampicillin resistance.

FBW7–SKP1 and FBW7^A677I^–SKP1 were produced following the same protocol. A 10 mL pre‐culture of transformed Rosetta (DE3) cells were used to inoculate a 1 L culture of LB media supplied with 100 µg L^−1^ of ampicillin and 34 µg L^−1^ of chloramphenicol. The flask was incubated at 37 °C and 180 rpm until reaching an OD_600_ between 0.6 and 0.8. Temperature was then decreased at 18 °C and protein production was induced with 1 mm IPTG for 18 h. Cells were harvested by centrifugation at 3700 rpm and 6 °C for 30 min and the cell pellets were stored at –20 °C until use.

For protein purification, cell pellets were defrosted and resuspended in buffer A (50 mM Hepes pH 8.0, 200 mm NaCl, 5 mm DTT) supplemented with Pierce protease inhibitor cocktail (ThermoFisher Scientific). Cell lysis was carried out by sonication during 2 min at intervals of 10 s of sonication followed by 30 s of break on ice. Lysates were then clarified by double centrifugation at 4 °C and 8800 rpm for 30 min. Supernatant was filtered using 0.8 µm syringe filters (Sigma‐Aldrich).

ÄKTA start system (Cytiva) was used in all purification steps. The cleared lysate was applied to a 5 mL GSTrap 4B column (Cytiva) and washed with buffer A. Bound protein to the column was eluted with buffer B (50 mm Hepes pH 8.0, 200 mm NaCl, 20 mm Glutathione, 5 mm DTT). 50 U of thrombin protease was added to the eluted protein fraction, for the GST‐tag cleavage. The mixture was dialyzed overnight at 4 °C against buffer A using a 3.5 kDa dialysis membrane (Spectra/Por). The uncleaved protein was removed with a second GST purification step, performed as described above. The flow through was collected and dialyzed against Buffer C (50 mm Hepes pH 8.0, 50 mm NaCl) and then loaded onto 5 mL 5 mL Hitrap Heparin column (Cytiva). The protein was eluted with a NaCl gradient from 50 to 500 mm in buffer C and concentrated to 1 mg mL^−1^ for storage. The mass and purity were subsequently verified by SDS−PAGE and mass spectrometry (MALDI‐TOF). For crystallization, the FBW7‐SKP1 was further purified in a Superdex 200 (Cytiva) in 20 mm Tris‐HCl, pH 8.0, 200 mm NaCl, 5 mm dithiothreitol (DTT) and concentrated to 48 mg mL^−1^ by ultrafiltration.

### Chemicals

The parent molecules (MMX) and the different analogue molecules (AX‐MMX) were purchased from Molport, Life Chemicals Inc., Vitas‐M, ChemBridge, and Enamine Ltd., assuring a purity of more than >95% for all of them. In addition, a liquid chromatography‐mass spectrometry (LC‐MS) was performed to confirm the purity and stability of the molecule used for the biological assays, A5‐MMC17 (Data S4, Supporting Information).

### Surface Plasmon Resonance (SPR)

SPR experiments were carried out at 25°C using Biacore T200 SPR biosensor instrument (Cytiva). CM7 sensor chip (Cytiva) was inserted, preconditioned and normalized following the protocol recommended by the supplier (Cytiva). In all cases, immobilization was carried out using the standard amine coupling procedure. Upon NHS/EDC activation, the protein immobilization was performed at 5 µL min^−1^, using a 1:50 protein mixture diluted with 10 mm sodium acetate at pH 5.0. Once the immobilization level reached ≈7500 to 8500 RU, unreactive groups were blocked with 1m ethanolamine. Phosphate‐buffered saline (PBS: 10 mm phosphate pH 7.4, 150 mm NaCl) was used as an immobilization running buffer.

Interaction assays were performed in a buffer that consisted of 1.05x PBS, 0.05% Tween‐20, 0.5 mg mL^−1^ dextran and 5% DMSO. Initially, small‐molecules from virtual screening were tested at an initial 8‐point 2‐fold dilution series starting from 250 µm. The screening assays were performed at a flow rate of 60 µL min^−1^ and the ligand association and dissociation times were set to 60 and 120 s, respectively. Once there was an estimation of the *K*_d_ of the ligands, concentration range was adjusted for each ligand. The data was subtracted from the reference channel and corrected by the solvent correction ranging from 3% to 8% DMSO in the running buffer.

The Biacore T200 evaluation software 2.0 was used for data analysis. Background signals were corrected subtracting blank injections (blank subtraction) to the injected ligand signals. To estimate binding affinity, SPR data was fitted to a single interaction model, where steady state values were extracted from the sensorgrams recorded and plotted against the different concentrations assayed. If necessary, R_max_ was fixed considering the R_max_ expected from the amount of protein immobilized in the chip surface.

### SAR‐by‐Catalogue

An exhaustive analogue search was performed using the top SPR positive hits from the two VS campaigns as reference. Compounds with Tanimoto‐based similarities between 70% and 80% using MACCSkeys fingerprints were evaluated using the abovementioned VS docking and DUck filters. A final selection of 10 analogs was purchased to the respective providers.

### Crystallization and Structure Determination

For co‐crystallization experiments, the SKP1‐FBW7‐molecule complexes were prepared by mixing a 48 mg mL^−1^ solution of SKP1‐FBW7 with a twofold molar excess of various molecules in 25 mm Tris‐HCl, pH 8.0, 200 mm NaCl and 5 mm DTT. The complexes were crystallized in a solution containing 100 mm HEPES‐Na, pH 7.4, and 1.2 m Li_2_SO_4_ at 4 °C using the hanging‐drop vapor diffusion method. For soaking experiments, the SKP1‐FBW7 apo‐crystals were incubated with a fivefold molar excess of the molecules for more than 10 h at 4 °C prior to freeze. Crystals were flash‐frozen in solutions containing saturated Li_2_SO_4_. Diffraction data were collection at the beamlines 17‐ID‐1 and 17‐ID‐2 of National Synchrotron Light Source II (NSLS‐II). Data were processed using Fast DP.^[^ ^67^, ^68^ ^]^ The structures were determined by molecular replacement with the program MOLREP of the CCP4 suite, and the SKP1‐FBW7 structure (PDB ID: 2OVP) was used as the search model.^[^ ^48^ ^]^

### Microscale Thermophoresis (MST)

For protein labelling, Monolith Protein Labelling Kit RED‐NHS 2nd Generation (Nanotemper) was purchased and used following manufacturer's guidelines. FBW7–SKP1 and FBW7^A677I^–SKP1 were labeled with a fluorophore via amine coupling following the corresponding protocol provided by Nanotemper. Both proteins were diluted to obtain a concentration of ≈10 µm with the Labelling Buffer NHS (130 mm NaHCO_3_ pH 8.2–8.3, 50 mm NaCl). Dye RED‐NHS 2nd Generation was added with a 2.5‐fold excess of dye for labelling FBW7–SKP1 and with a 1‐fold excess of dye for labelling FBW7_A677I_–SKP. Protein concentration and its degree‐of‐labelling were calculated based on absorbance at 650 and 280 nm, measured with NanoDrop spectrophotometer (Thermo‐Scientific)

MST experiments were performed on Monolith NT.115 (Nanotemper) at 25°C. Monolith NT.115 Premium Capillaries (Nanotemper) were used. LED (light emitting diode) power was set to 40% or 60%, while MST power was set at 20% and 40%. All the experiments were carried out in triplicates in a buffer that contained 50 mm Hepes pH 8.0, 50 mm NaCl, 0.05% Tween‐20 and 1 mm DTT (MST buffer). Samples were prepared at a final volume of 20 µL and the final concentration of the labeled protein was 50 nm. A5‐MMC17 was tested at 12‐point 1:2 dilution starting at 1 mm. The results were analyzed using MO. Affinity Analysis v2.3 (Nanotemper). Data was evaluated using initial fluorescence mode, where raw fluorescence counts were plotted against ligand concentration to obtain the *K*_d_. To discriminate if initial fluorescence was caused by the ligand interaction with the protein or caused by sample loss due to aggregation or surface adsorption, SD‐test was carried out following manufacturer's recommendations. The three highest and lowest concentrations of ligand‐protein mixture were centrifuged at 15000 g for 10 min to remove precipitates. Protein denaturation was induced by adding 1 volume of 4% SDS and 40 mm DTT followed by an incubation at 95 °C for 5 min. Upon measurement, if no differences in initial fluorescence were observed between low and high ligand concentrations after SD‐test, it was concluded that the initial fluorescence change was induced by a binding event.

### Isothermal Titration Calorimetry (ITC)

ITC experiments were performed using Nano ITC Low Volume (TA Instruments). All titrations were carried out at a temperature of 25 °C. DMSO concentrations were the same in the sample cell with the protein solution and in the syringe with the ligand, which were filled at final volumes of 320 and 50 µL, respectively. The reference cell was filled with 320 µL of milli‐Q water (MQW). FBW7–SKP1 and FBW7_A677I_–SKP1 were buffer exchanged in 50 mm HEPES pH 8.0, 50 mm NaCl. In all experiments an initial injection of 0.48 µL was performed followed by 20 identical injections of 2.5 µL. 30 µm FBW7–SKP1 solution was titrated against 600 µm of A5_MMC17 at 5% DMSO. To compare their binding affinities, 30 µm FBW7_A677I_–SKP1 solution was titrated against 600 µm of A5‐MMC17 at 5% DMSO. The heat of dilution was determined by carrying out the same experiment with a buffer instead of protein in the sample cell and was subtracted from the experimental data.

Data was analyzed using the NanoAnalyze^TM^ software (TA Instruments) to directly obtain the enthalpy of binding (∆H) and the binding constants (*K*_a_ and *K*_d_). A single binding site model was applied, and the first data point was excluded from the analysis. From that, thermodynamic parameters were calculated using the Gibbs free energy equation.

### Cell Viability Assays

HEK293 cells were seeded at 80%–90% confluency in 96‐white well plates (CORNING). Cells were treated with the different molecules in triplicate, at 10, 100, and 250 µm. After 2h treatment, CellTiter‐Glo reagent (Promega) was prepared and added in a proportion 1:1 to the plate. The plate was shaken at 200 rpm for 2 min, and incubated at RT for 10 min, before the luminescence was measured at 562 nm in a CLARIOstar microplate reader (BMG Labtech).

### Immunoblotting

HEK293, HCT116 FBW7^WT^ and HCT116 FBW7^‐/‐^ cells were purchased from ATCC, and Cultek respectively and were grown in DMEM (HEK293) or RPMI‐1640 (HCT116) supplemented with 10% Fetal Bovine Serum (FBS), 2 mm L‐Glutamine and 100 U µg^−1^ penicillin‐streptomycin at 37 °C in a humidified atmosphere of 5% CO2. Cells were treated for periods of 2 or 4h with the different compounds tested. The co‐treatments with the different inhibitors assessed were added 1h before compound addition. The proteasome inhibitor (MG‐132) (Merck) was added at 10 µm and the neddylation‐activating enzyme (NAE) inhibitor (MLN4924) (Merck) was added at 3 µm. After compound treatment, cells were washed twice with PBS 1 and lysed with RIPA lysis buffer (Thermo Fisher) supplemented with protease inhibitors (Thermo Fisher). Samples were incubated on ice for 10 min and lysates were clarified by 20 min centrifugation at 14000 rpm, at 4°C. The total protein was quantified using a BCA commercial kit (ThermoFisher), following the manufacturer's instructions. Normalized amounts of protein were loaded into 10% Tris/Gly/SDS gels and resolved at constant intensity (25 mA gel^−1^). The resolved proteins were electrotransferred onto PVDF membranes (iBlot2 system, Invitrogen). Then, the membranes were blocked with TBST+5% non‐fat dry milk 1h at RT and probed with the corresponding antibodies. The membranes were probed using the chemiluminescence (ECL) western blotting substrate (Thermo Fisher Scientific) in an Amersham Imager 680 System (Cytiva). The images obtained were analyzed using Image Lab software (BioRad).

### Statistical Information

The statistical analysis was conducted using GraphPad Prism 10.0 software. Student t‐test, one‐way ANOVA or two‐way ANOVA were employed to determine the statistical significance of the comparisons. Differences were considered statistically significant when *P* < 0.05. *P*‐values were denoted by asterisks as follows: (^*^) *P* < 0.05; (^**^) *P* < 0.01; (^***^) *P* < 0.001; (^****^) *P* < 0.0001.

## Conflict of Interest

C.G. and X.B. are co‐founders and shareholders of Oniria Therapeutics. C.G. is drug discovery consultant of Oniria Therapeutics.

## Author Contributions

M.M.‐C., A.S.‐M., and A.B.‐M. contributed equally to this work. M.M‐C. performed and analyzed computational and biophysical experiments. A.S.‐M. designed, performed and analyzed the computational workflow. Wrote the manuscript. A. B.‐M.^:^ performed biophysical and biological experiments. Wrote the manuscript. R.C.‐M. designed and performed single‐point mutation experiments. S.S. performed biophysical experiments. V.I. performed and analyzed computational experiments. N.C. performed PhotoXplorer screening. J.S. performed LC‐MS/MS measurements. P.A‐B. managed and analyzed the PhotoXplorer experiments. G.M.K. Designed and supervised and analyzed the PhotoXplorer experiments. Wrote the manuscript. Funding acquisition. Y.L. designed, performed and analyzed the X‐ray experiments. B.H.^:^ designed, performed and analyzed the X‐ray experiments. X.B. conceptualization of the project, designed the computational approach, resources, and acquired funding acquisition. C.G. conceptualization of the project, led the project, resources, and acquired funding. Wrote the manuscript.

## Supporting information



Supporting Information

## Data Availability

The data that support the findings of this study are openly available in Zenodo at https://10.5281/zenodo.14878508, reference number 14878509.
