# Supplementary material for: Discovering Uncharted Binding Pockets on E3 Ligases Leads to the Identification of FBW7 Allosteric Modulators
Source: Adv Sci (Weinh). 2025 Aug 20;12(39):e06068. doi: 10.1002/advs.202506068 (PMC12533291; doi:10.1002/advs.202506068)

ADVANCED SCIENCE
Open Access

Supporting Information

for *Adv. Sci.*, DOI 10.1002/advs.202506068

Discovering Uncharted Binding Pockets on E3 Ligases Leads to the Identification of FBW7 Allosteric Modulators

*Míriam Martínez-Cartró, Álvaro Serrano-Morrás, Andrea Bertran-Mostazo, Roger Castaño-Muñiz, Salvatore Scaffidi, Varbina Ivanova, Noémi Csorba, József Simon, Péter Ábrányi-Balogh, Yunfeng Li, György M. Keserü, Bing Hao, Xavier Barril and Carles Galdeano**

Supporting Information for

Discovering Uncharted Binding Pockets on E3 Ligases Leads to the Identification of FBW7 Allosteric Modulators

Míriam Martínez-Cartró[1,2#], Álvaro Serrano-Morras[1,2#], Andrea Bertran-Mostazo[1,2#], Roger Castaño-Muñiz[1,2], Salvatore Scaffidi[1,2], Varbina Ivanova[1,3], Noémi Csorba[4,5,6], József Simon[4,5], Péter Ábrányi-Balogh[4,5,6], Yunfeng Li[7], György M. Keserü[4,5,6], Bing Hao[7], Xavier Barril[1,2,3,8*] and Carles Galdeano[1,2*]

[1]Departament de Farmàcia i Tecnologia Farmacéutica, i Fisicoquímica. Facultat de Farmàcia i Ciències de l'Alimentació Universitat de Barcelona, Barcelona, 08028, Spain
E-mail: cgaldeano@ub.edu

[2]Institut de Biomedicina de la Universitat de Barcelona (IBUB), Universitat de Barcelona, Barcelona, 08028, Spain

[3]Institut de Química Teòrica i Computacional (IQTC), Universitat de Barcelona, Barcelona, 08028, Spain

[4]Medicinal Chemistry Research Group. HUN-REN Research Center for Natural Sciences, Budapest, 1117, Hungary

[5]National Drug Research and Development Laboratory. HUN-REN Research Center for Natural Sciences, Budapest, 1117, Hungary

[6]Department of Organic Chemistry and Technology. Budapest University of Technology and Economics, Budapest, 1117, Hungary

[7]Department of Molecular Biology and Biophysics. University of Connecticut School of Medicine, Farmington, CT, 06030, USA

[8]Catalan Institution for Research and Advanced Studies (ICREA), Barcelona, 08010, Spain

[#]These authors contributed equally to this work.

The PDF file includes:

Supporting Figures (page 2)
Supporting Data (page 22)

SUPPORTING FIGURES

**Figure S1. Dendrograms illustrating the classification of complex (a) and simple (b) E3 ligases.**	page 3
**Figure S2. Table of the 23 E3 ligases selected for the ligandability study.**	page 4
**Figure S3. Table summarizing the results of the MDMix ligandability study.**	page 6
**Figure S4. Homology study for the different FBW7 ortholog genes.**	page 9
**Figure S5. Validation of FBW7 pockets by PhotoXplorer screening technology.**	page 10
**Figure S6. Virtual screening workflow followed for the identification of FBW7 ligands in FBW7-Pocket G.**	page 11
**Figure S7. Table of the positive binders identified by SPR.**	page 12
**Figure S8. Heatmap of the structural stability prediction of the A677I mutant using the FoldX software.**	page 15
**Figure S9. Results comparing the binding of A5-MMC17 to FBW7WT-SKP1 (black) and FBW7A677I-SKP1 (red).** (a) ITC results (b) SPR results	page 16
**Figure S10. FBW7–SKP1 X-ray structure.**	page 18
**Figure S11. Immunoblotting results of 4h of A8-MMC37 treatment in HEK293.**	page 19
**Figure S12. Immunoblotting results of other FBW7 substrates after 4h of A5-MMC17 treatment.**	page 20

Figure S1. **Dendrograms illustrating the classification of complex (a) and simple (b) E3 ligases.** The selected E3 ligases for the ligandability study are denoted in colour. Pictures modified from Ubihub.[18]

(a)

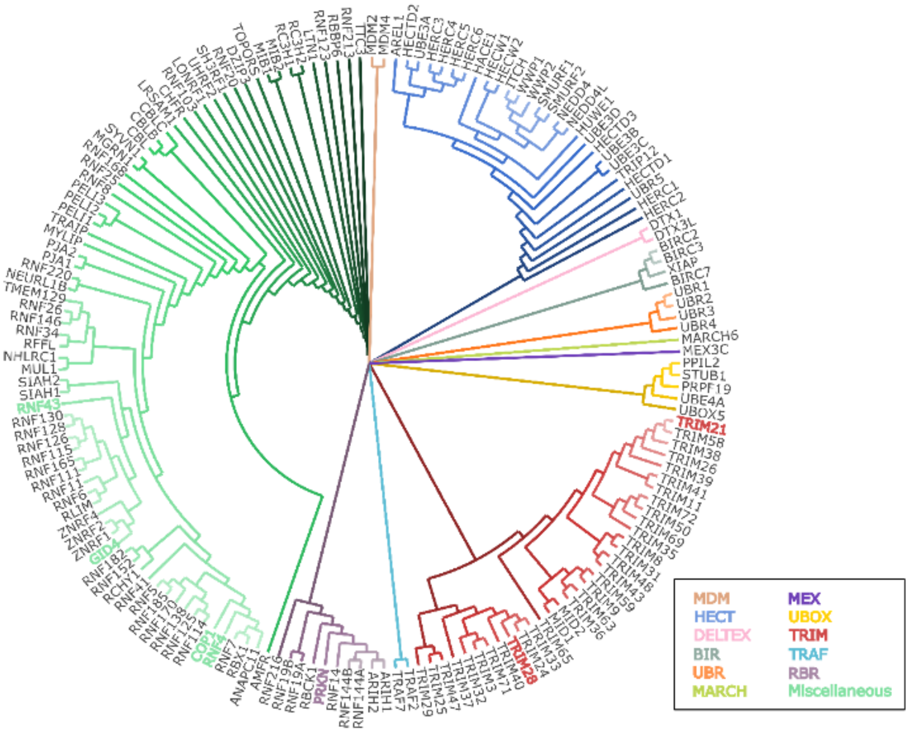

(b)

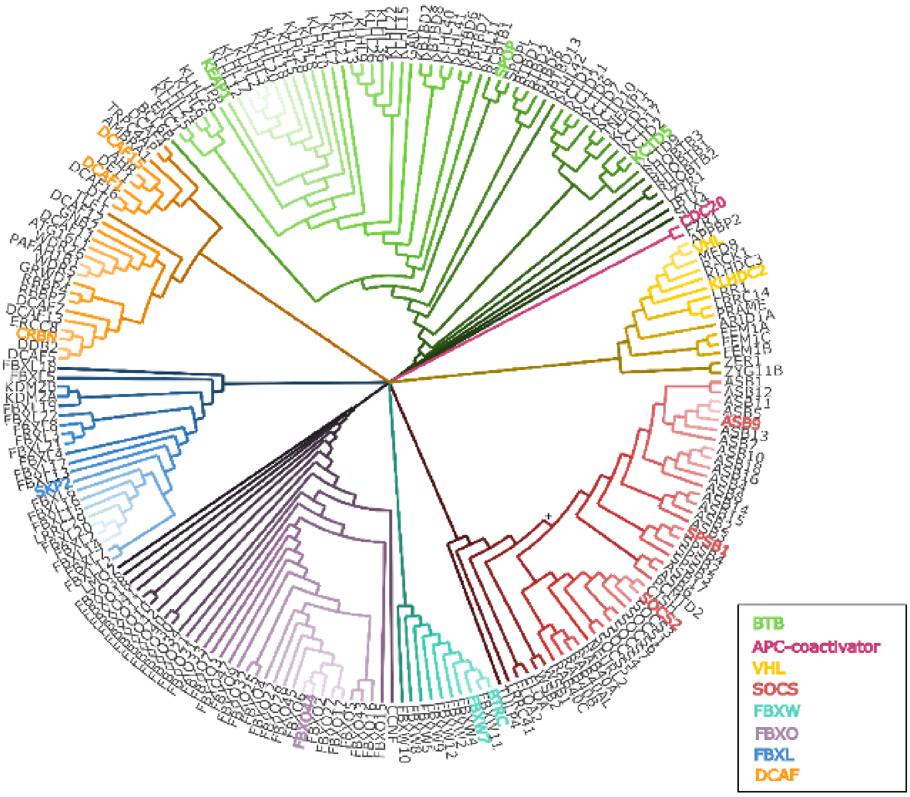

Figure S2. **A) Table of the 23 E3 ligases selected for the ligandability study. B) Plot comparing resolution versus coverage for all E3 ligases with all available X-ray structures (as listed in the UbiHub database) alongside our selected E3 ligases**

A)

E3	PDB id (aa range)	Substrate Recognition domain (aa range)	Specific Coverage	General Coverage	Resolution (Å)
ASB9	3ZKJ (35-294)	ANK1-3 (35-130)	1	0.88	1.58
BTRC	6M90 (175-605)	WDR (301-590)	1	0.66	2.05
CDC20	4GGC (161-477)	WDR (182-480)	0.99	0.63	1.35
CRBN	5FQD (41-442)	CULT (318-426)	1	0.85	2.45
COP1	5HQG (376-731)	WDR (419-677,691-729) & TRIB1 interaction domain (643-645)	1	0.44	2.00
DCAF1	4CC9 (1058-1396)	WDR (1091-1290)	1	0.2	2.47
DCAF15	6UD7 (2-600)	RBM39 binding motif (230-480)	1	0.71	2.30
FBO44	3WSO (1-255)	FBA (71-252)	1	0.99	1.60
FBW7	2OVP (263-707)	WDR (378-659)	1	0.62	1.60
GID4	6CCU (116-300)	N-degron recognition domain (120-280)	1	0.55	1.75
KCTD5	3DRX (34-234)	BTB/POZ (44-146)	1	0.76	2.11
KEAP1	6TYM (321-609)	KELCH (327-611)	0.99	0.46	1.42
KLHDC2	6DO3 (1-362)	KELCH (31-359)	1	0.78	2.17
PRKN	5C1Z (1-465)	SYT11 binding domain (204-293)	1	0.83	1.79
RNF4	4PPE (120-190)	TRPS1/SUMO interaction domain (4-61) & Zinc Finger (132-177)	0 & 1	0.35	2.00
RNF43	4KNG (44-198)	-	-	0.19	2.50
SKP2	2AST (89-424)	LRR (151-401)	1	0.77	2.30
SOCS2	6I5N (30-198)	SH2 (48-156) & SOCS-box (151-197)	1	0.81	1.98
SPOP	3HQI (28-329)	MATH (31-161)	1	0.78	1.62
SPSB1	2JK9 (30-231)	SPRY (33-231)	1	0.74	1.79
TRIM21	2IWG (287-465)	SPRY (268-465)	0.9	0.38	2.35
TRIM28	6QAJ (56-413) & 2RO1 (624-812)	RBCC (65-376) & PHD (625-672)	1 & 1	0.33 & 0.23	1.90
VHL	4W9H (54-213)	VHL-beta (12-93) & VHL-box (105-153)	0.48 & 1	0.74	2.1

B)

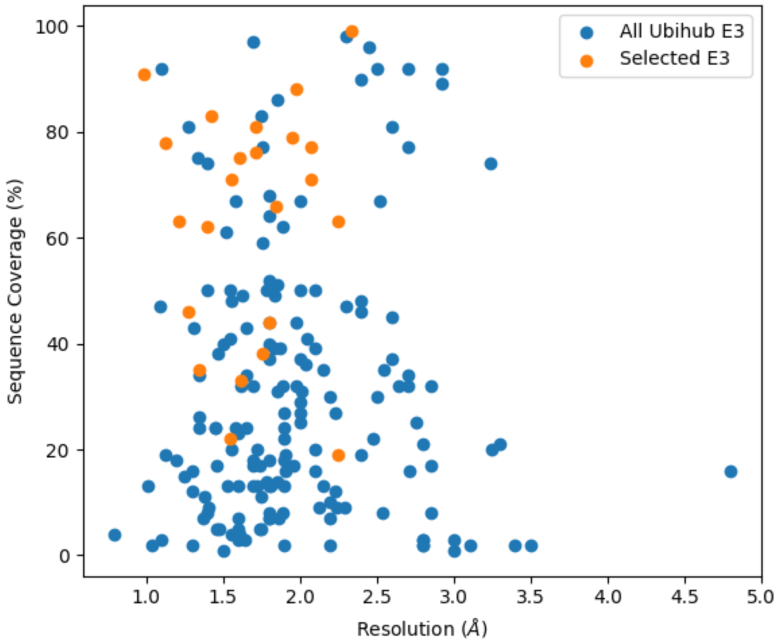
All Ubihub E3
Selected E3
Sequence Coverage (%)
Resolution (Å)
100
80
60
40
20
0
1.0
1.5
2.0
2.5
3.0
3.5
4.0
4.5
5.0

Figure S3. **Table summarizing the results of the MDMix ligandability study.** The table contains the energy values and the description of the hotspot of the identified ligandable pockets.

E3 ligase	Pocket	ΔG_{bind} (kcal/mol)	Hotspots (Polar/Hydrophobic)	Pocket efficency ΔG_{bind}/Nº hotspots (kcal/mol)	Location
ASB9	G	-4.61	4 (3/1)	-1.15	Allosteric
	H	-4.12	4 (3/1)	-1.03	Allosteric
BTRC	A	-9.7	9 (6/3)	-1.08	Allosteric
	C	-6.42	6 (4/2)	-1.07	Degron
	D	-8.4	8 (5/3)	-1.05	Allosteric
CDC20	A1	-5.96	4 (2/2)	-1.49	Allosteric
	E	-5.5	4 (1/3)	-1.38	Allosteric
COP1	D	-9.29	8 (4/4)	-1.16	Degron
CRBN	B	-12.04	11 (5/6)	-1.09	Allosteric
	E	-6.18	6 (3/3)	-1.03	Allosteric
	G	-5.65	5 (3/2)	-1.13	Ligand
	J	-9.82	9 (5/4)	-1.09	Allosteric
DCAF1	A	-14.4	11 (4/7)	-1.31	Degron
	D	-8.63	7 (4/3)	-1.23	Allosteric
	J	-5.34	5 (2/3)	-1.07	Allosteric
DCAF15	B	-12.8	11 (6/5)	-1.16	Allosteric
	F	-6.18	5 (3/2)	-1.24	Degron
	H	-9.47	8 (2/6)	-1.18	Allosteric
FBO44	B	-9.15	9 (3/6)	-1.02	Allosteric
FBW7	B	-7.6	8 (5/3)	-0.95	Degron
	D	-5.31	5 (2/3)	-1.06	Allosteric
	G	-8.76	8 (5/3)	-1.13	Allosteric
GID4	A	-6.47	5 (3/2)	-1.29	Allosteric

	B	-6.03	5 (3/2)	-1.21	Allosteric
KCTD5	A1	-6.46	5 (3/2)	-1.29	Allosteric
	E	-6.98	6 (3/3)	-1.16	Allosteric
	H	-4.65	4 (2/2)	-1.16	Allosteric
KEAP1	B	-9.61	8 (5/3)	-1.20	Allosteric
	D	-10.14	8 (3/5)	-1.27	Degron
KLHDC2	B	-10.42	8 (4/4)	-1.30	Degron
	E	-8.5	6 (5/1)	-1.42	Allosteric
PRKN	A	-9.2	11 (5/6)	-0.84	Allosteric
	C	-8.43	9 (5/4)	-0.94	Degron
RNF4	-	-	-	-	-
RNF43	A	-8.76	7 (4/3)	-1.25	Allosteric
	C	-6.48	6 (4/2)	-1.08	Degron
	E	-4.98	4 (3/1)	-1.25	Degron
	F	-7.75	6 (4/2)	-1.29	Allosteric
SKP2	D	-10.08	11 (5/6)	-0.92	Degron
SOCS2	A	-4.83	4 (2/2)	-1.21	Allosteric
SPOP	C	-7.63	7 (4/3)	-1.09	Allosteric
SPSB1	B	-6.92	5 (3/2)	-1.38	Allosteric
	C	-6.66	6 (3/3)	-1.11	Allosteric
TRIM21	A	-6.43	5 (3/2)	-1.29	Allosteric
	C	-5.93	5 (2/3)	-1.19	Degron
	D	-5.27	4 (2/2)	-1.32	Allosteric
TRIM28	RBCC-A	-18.657	16 (8/8)	-1.17	Allosteric
	RBCC-B	-13.284	11 (5/6)	-1.21	Allosteric
	RBCC-K	-9.162	9 (4/5)	-1.02	Allosteric
	PHD-BR-A	-7.280	8 (4/4)	-0.91	Allosteric
	PHD-BR-C	-5.580	5 (2/3)	-1.12	Allosteric

VHL	B	-6.68	7 (4/3)	-0.95	Allosteric
	C	-9.6	9 (4/5)	-1.07	Allosteric
	E	6.26	6 (3/3)	1.04	Degron

***Figure S4*. Homology study for the different FBW7 ortholog genes.** The results show a conserved region in all the ortholog genes in the FBW7-pocketG sequence regions. Colours correspond to ClustalX nomenclature. The alignment was performed with ClustalW and the visualization with Jalview.

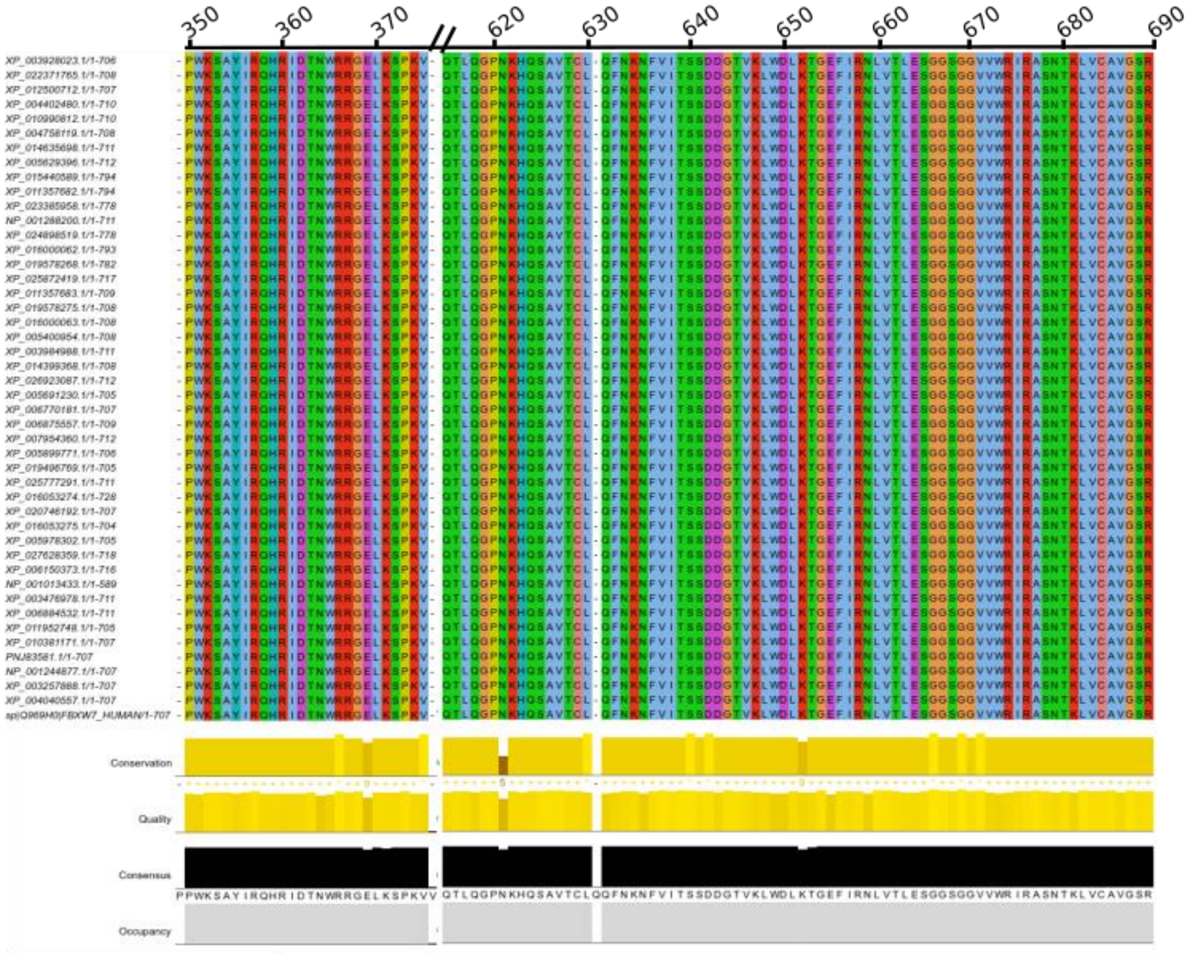

Figure S5. **Validation of FBW7 pockets with PhotoXplorer.** (a) Fbw7 Php fragment hits and their occupancy levels measured by intact MS. (b) Php-labelled residues of the three most promising compounds from the fragment screening determined by tryptic digestion and LC-MS/MS. (c) Identified stable binding mode of Php013 in the covalently bound Fbw7 complex (Php013 in green, labelled cysteine residue in cyan). (d) RMSD of the predicted binding mode of Php013 along 5 replicas of free MD simulations.

(a)

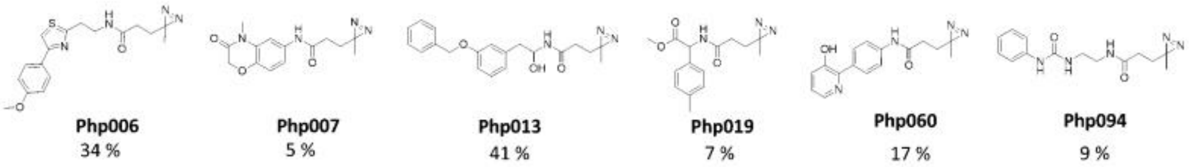

(b)

Compound	**Php006**	**Php013**	**Php060**
Labeled residue	$Fbw7^{Q306}$, $Fbw7^{C308}$	$Fbw7^{C390}$	$Fbw7^{C613}$ and $Fbw7^{T307}$, $Fbw7^{C308}$, $Fbw7^{R309}$

(c) **(d)**

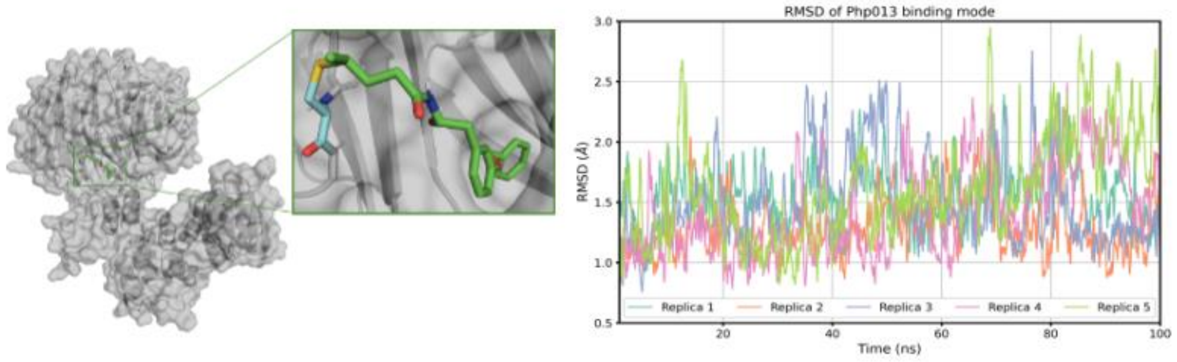

Figure S6. **Virtual screening workflow followed for the identification of FBW7 ligands in FBW7-pocket G.**

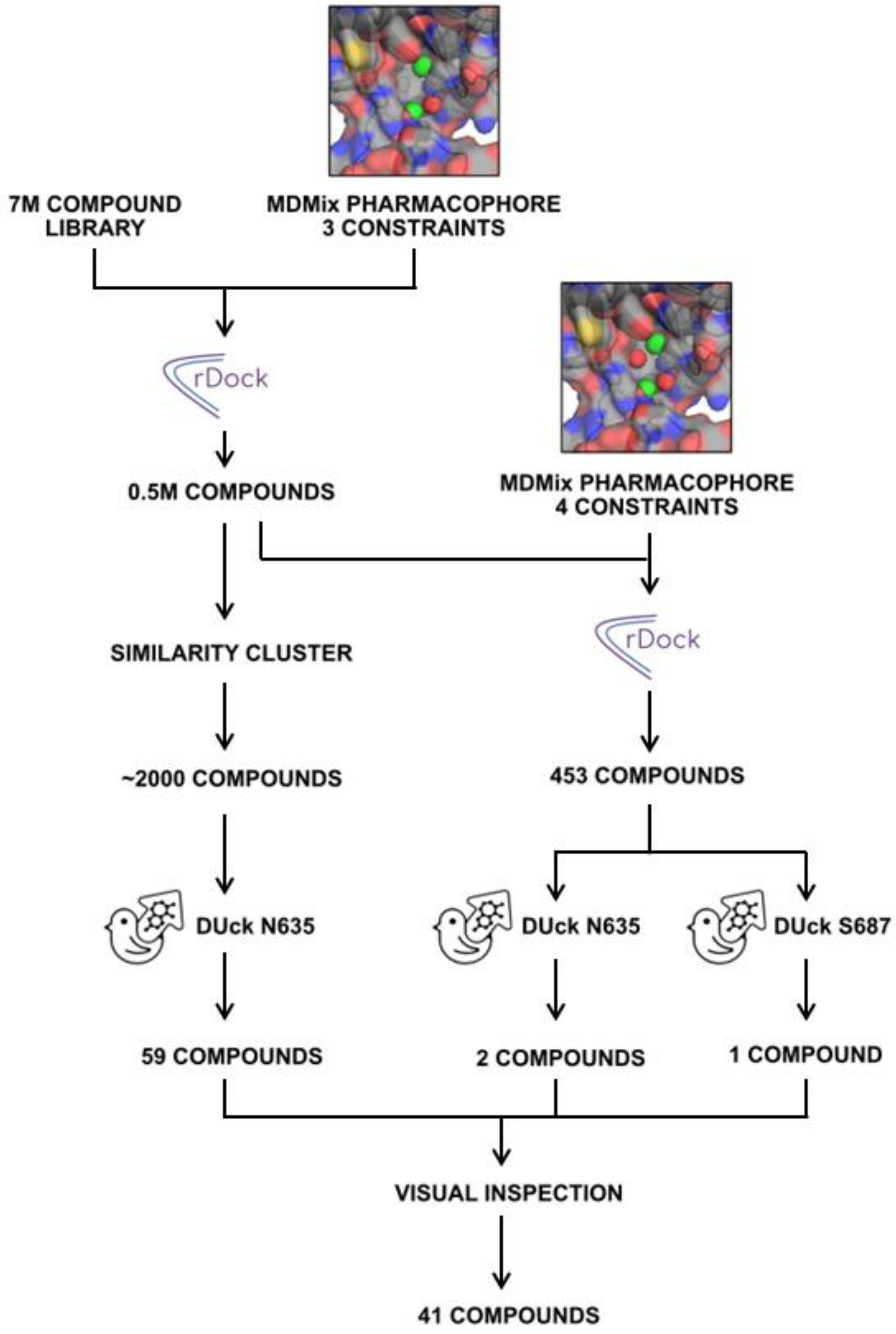

Figure S7. **Table of the positive binders identified by SPR**. The table contains the molecule name, the structure and the K_d obtained by SPR.

Molecule	Analogue of	Structure	SMILES	Vendor ID	K_D (µM)
A9-MMC37	MMC37	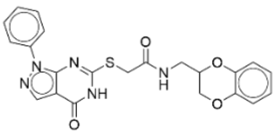	O=C(CSC1=Nc2c(cnn2-c2ccccc2)C(=O)N1)NCC1CO c2ccccc2O1	Z50928 046	2
MMC40	-	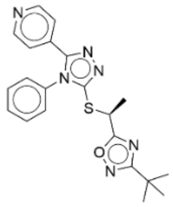	C[C@H](Sc1nnc(-c2ccncc2)n1-c1ccccc1)c1nc(C(C)(C)C)no1	Z33573 9488	2
A8-MMC37	MMC37	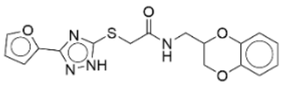	O=C(CSc1nc(-c2ccco2)n[nH]1)NCC1COc2c cccc2O1	Z22055 9878	23
A3-MMC21	MMC21	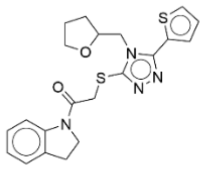	O=C(CSc1nnc(-c2cccs2)n1CC1CCCO1)N1C Cc2ccccc21	Z19524 536	41
A5-MMC17	MMC17	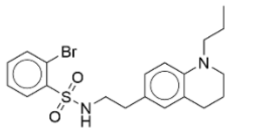	CCCN1CCCc2cc(CCNS(=O)(=O)c3ccccc3Br)ccc21	F2294-0112	45
MMC21	-	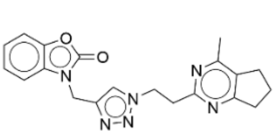	Cc1nc(CCn2cc(Cn3c4ccccc4 oc3=O)nn2)nc2c1CCC2	315903 44	51
A6-MMC17	MMC17	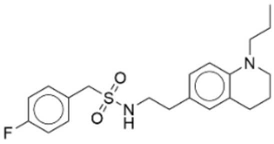	CCCN1CCCc2cc(CCNS(=O)(=O)Cc3ccc(F)cc3)ccc21	F2294-0170	60

MMC4	-	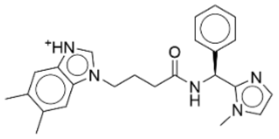	Cc1cc2[nH+]cn(CCCC(=O)N[C@H](c3nccn3C)c3ccccc3)c2cc1C	Z445006390	63
MMC17	-	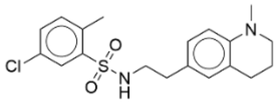	CN1CCCc2cc(CCNS(=O)(=O)c3cc(Cl)ccc3C)ccc21	F2294-0072	71
MMC2	-	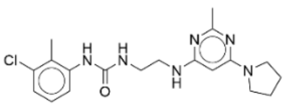	Cc1nc(NCCNC(=O)Nc2cccc(Cl)c2C)cc(N2CCCC2)n1	9238231	76
A2-MMC11	MMC11	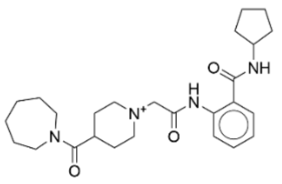	O=C(C[N+]1CCC(C(=O)N2CCCCCC2)CC1)Nc1ccccc1C(=O)NC1CCCC1	Z106748400	127
MMC11	-	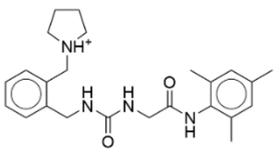	Cc1cc(C)c(NC(=O)CNC(=O)NCc2ccccc2C[NH+]2CCCC2)c(C)c1	Z211896442	135
MMC42	-	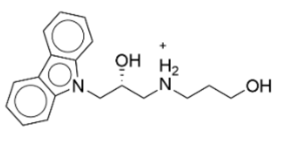	OCCC[NH2+]C[C@@H](O)Cn1c2ccccc2c2ccccc12	STK056081	135
MMC35	-	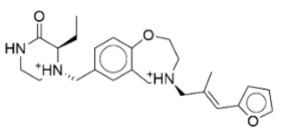	CC[C@H]1[N@H+](Cc2ccc3c(c2)C[N@H+](C/C(C)=C/c2ccco2)CCO3)CCNC1=O	75590452	136
A1-MMC11	MMC11	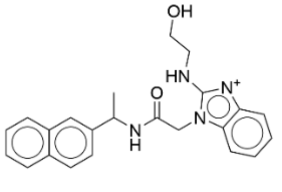	CC(NC(=O)Cn1c(NCCO)[n+]c2ccccc12)c1ccc2ccccc2c1	Z25714666	139

A4-MMC17	MMC17	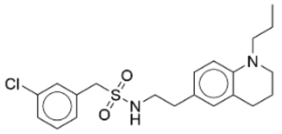	CCCN1CCCc2cc(CCNS(=O)(=O)Cc3cccc(Cl)c3)ccc21	F2294-0172	140
MMC37	-	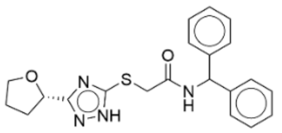	O=C(CSc1nc([C@@H]2CCCO2)n[nH]1)NC(c1ccccc1)c1ccccc1	Z221562842	234
A7-MMC2	MMC2	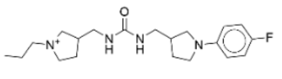	CCC[N+]1CCC(CNC(=O)NCC2CCN(c3ccc(F)cc3)C2)C1	Z821008348	280
A10-MMC40	MMC40	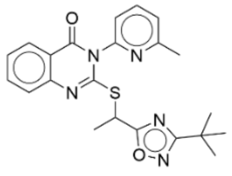	CC(SC1=Nc2ccccc2C(=O)N1c1cccc(C)n1)c1nc(C(C)(C)C)no1	Z298058550	353

Figure S8. **Heatmap of the structural stability prediction of the FBW7^{A677I} mutant using the FoldX software.** The FoldX pipeline predicts the free energy of unfolding and represents in the colour scale the ΔΔG of single point mutations. On the x axis there is the protein sequence and their respective mutation to all the possible residues on the y axis. FBW7^{A677I} mutant (AA2677 on the heatmap) was chosen as having little impact on the protein folding stability.

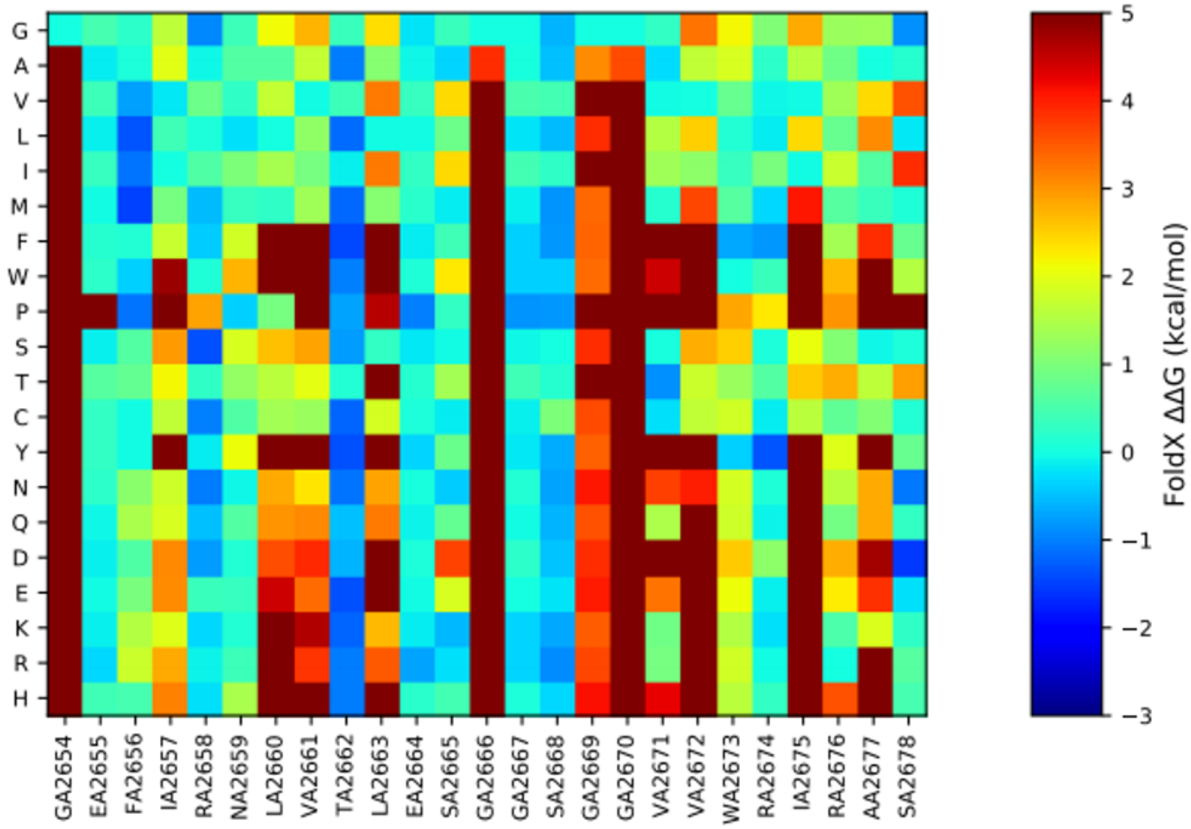

Figure S9. **Results comparing the binding of A5-MMC17 to FBW7^{WT}-SKP1 (black) and FBW7^{A677I}-SKP1 (red).** (a) ITC results show a clear binding of A5-MMC17 to FBW7^{WT}-SKP1, whereas a remarked reduction in the affinity when binding to FBW7^{A677I}-SKP1. Results correlate with the ones observed in different orthogonal techniques.

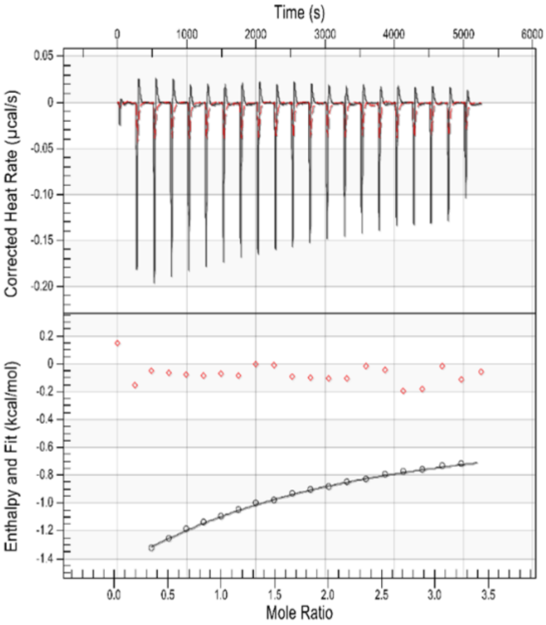

ITC results of A5_MMC17 binding to FBW7–SKP1

Model	Variable	Value	CI
Independent	K_d (µM)	50.900	6.007
	n	1	Fixed
	ΔH (kcal/mol)	-2.052	±0.140
	ΔS (cal/mol·K)	12.76	
	ΔG (kcal/mol)	-5.85	
Blank (linear)	Intercept (µcal)	-1	Fixed
	Slope	0.008	±0.001
	Confidence Level		90%

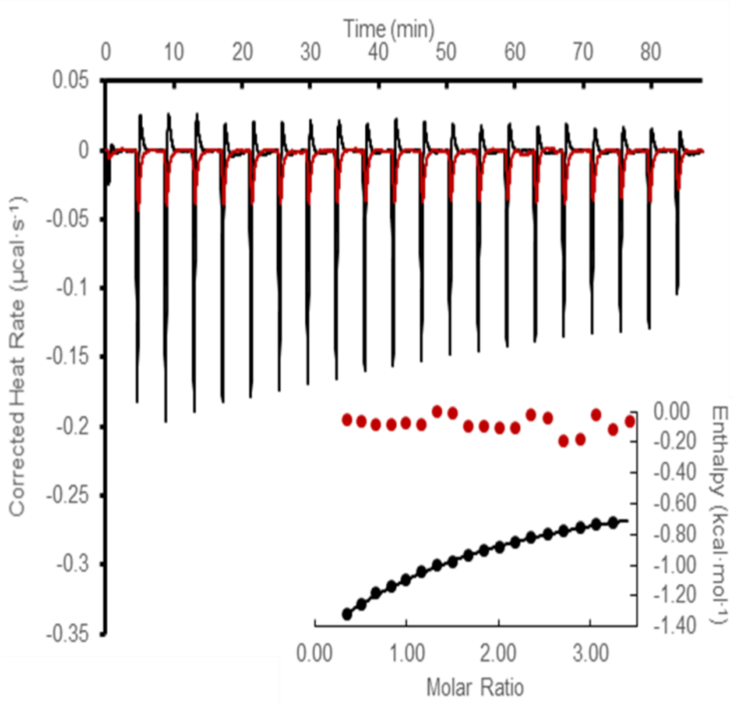

(b) SPR results show a clear binding of A5-MMC17 to FBW7^{WT}-SKP1, whereas a remarked reduction in the affinity when binding to FBW7^{A677I}-SKP1. Results correlate with the ones observed in different orthogonal techniques.

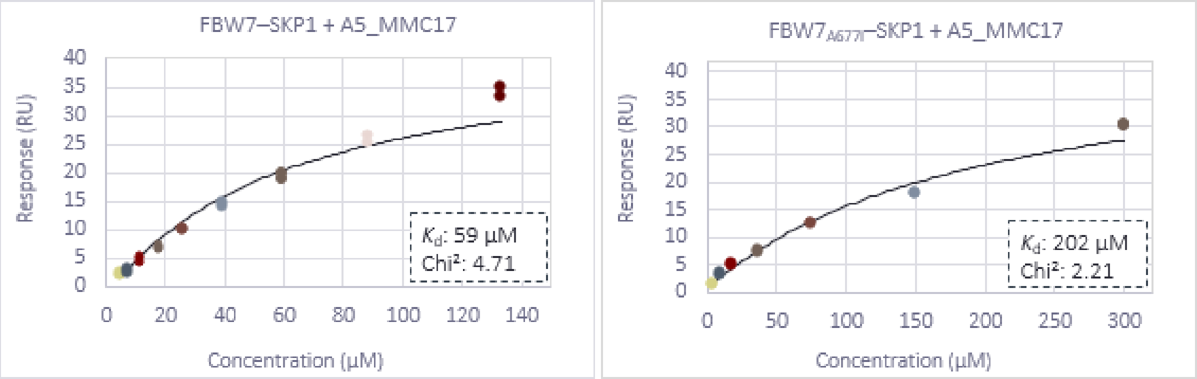

Figure S10. **The calculated electron density map for the structure of the SKP1-FBW7 crystals soaked with MMC17.** The protein is shown in stick with the corresponding 2Fo-Fc density map (contoured at 1 σ) coloured in blue. The additional Fo-Fc densities (contoured at 3 σ) that correspond to the sulphate ion were shown in green.

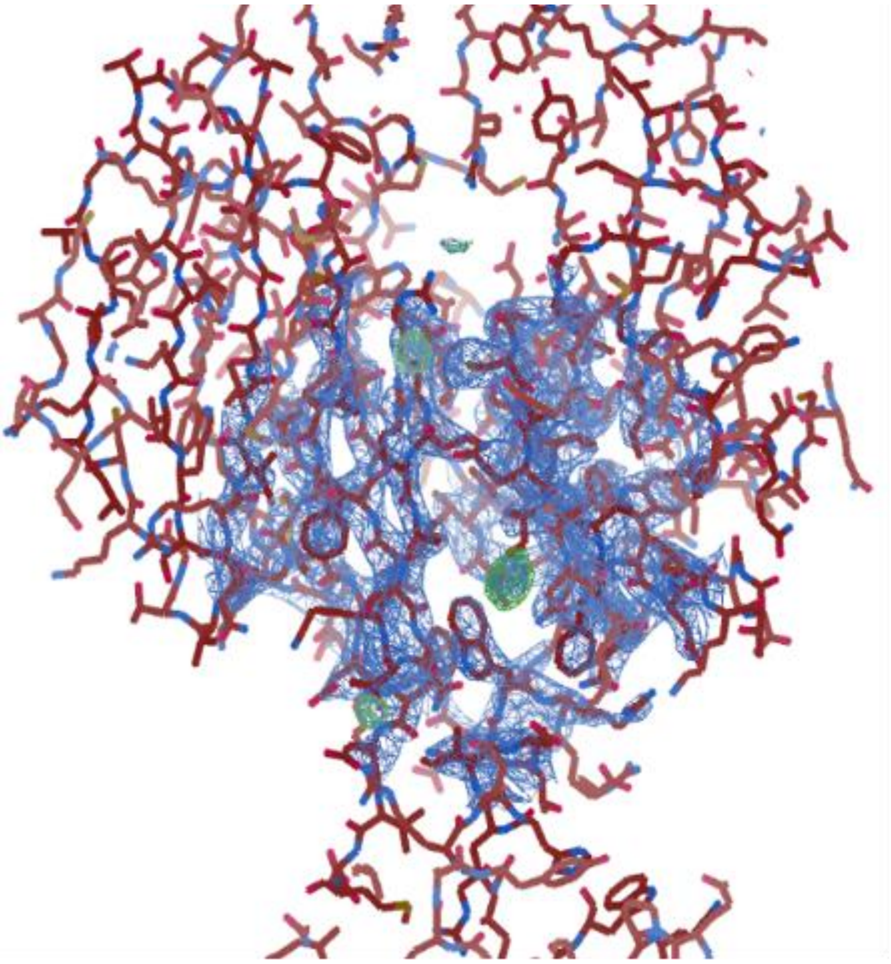

Figure S11. **Immunoblotting results of 4h of A8-MMC37 treatment in HEK293**. Results show that A8-MMC37 seems to be a silent binder, since it demonstrated to not cause a reduction in cell viability and to not trigger a reduction of c-Myc. At least three independent replicates for each condition (n=3).

- WB c-MYC/c-JUN degradation molecule A8-MMC37 (no reduction in cell viability, no degradation c-Myc)

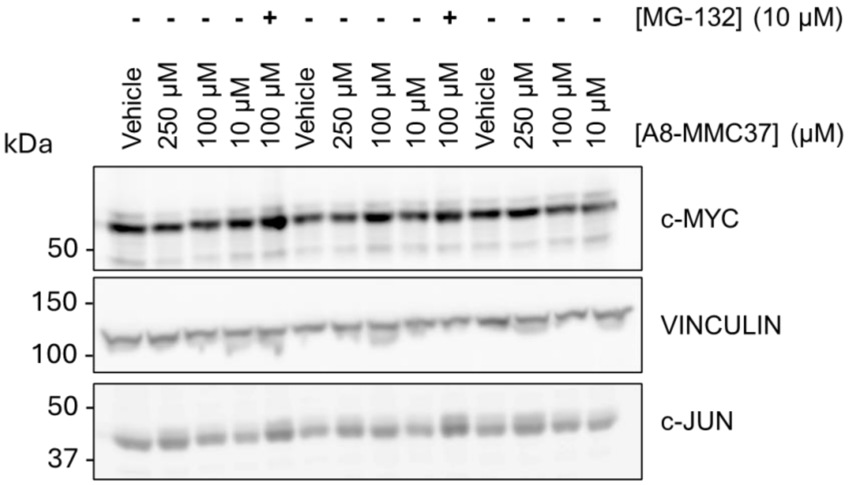

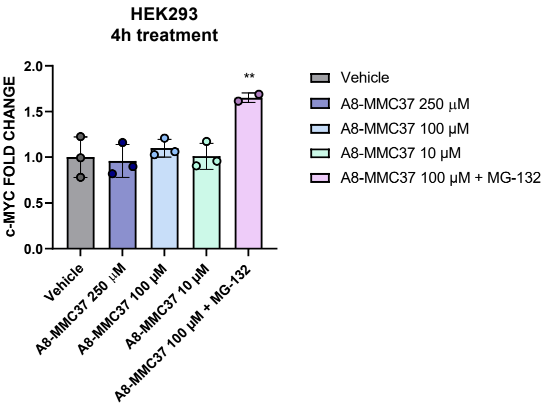

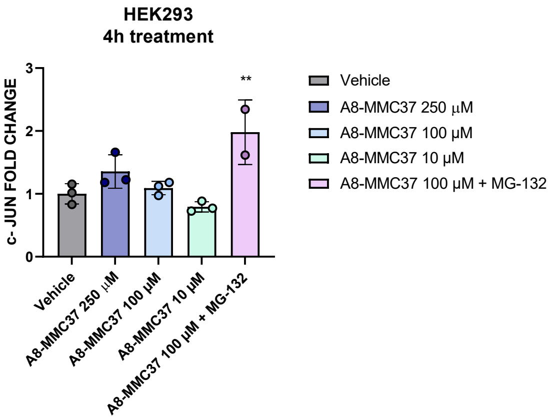

***Figure S12*. Immunoblotting results of other FBW7 substrates after 4h of A5-MMC17 treatment.**

(a) Immunoblotting results showing the on-target effect resulting in a dose-dependent c-Jun degradation. Results show a reduction of c-Jun after A5-MMC17 treatment in FBW7^{WT} cells (blue), whereas it has a silent effect in FBW7$^{-/-}$ cells (yellow). At least two independent replicates for each condition (n=3).

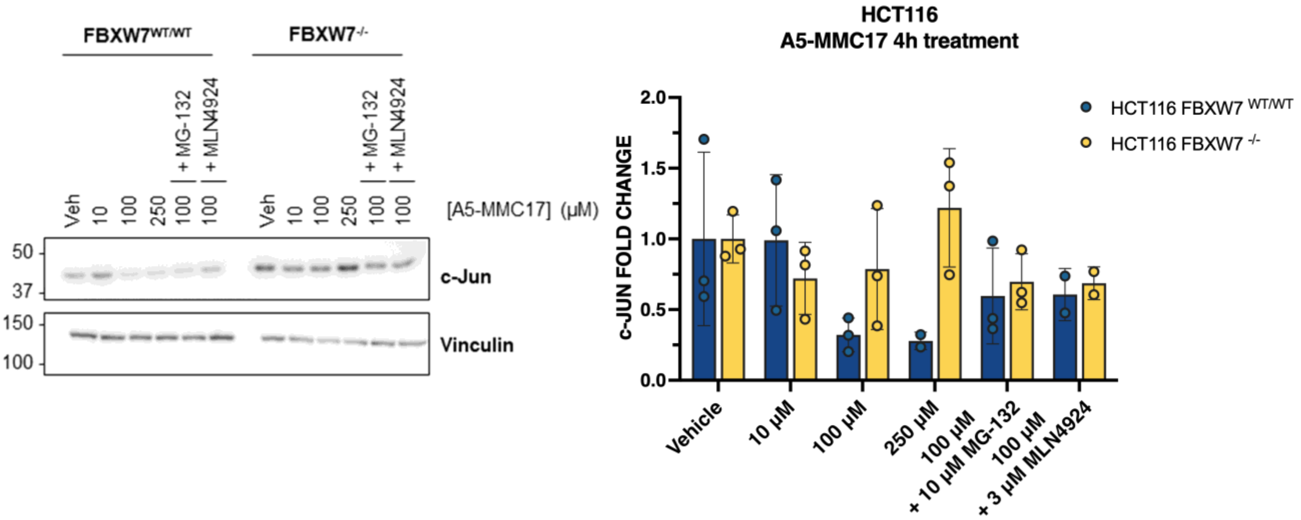

(b) Immunoblotting results showing that 4h of A5-MMC17 treatment did not induce a reduction of other FBW7 substrates: **CCNE1, MCL-1 and Notch1**. At least two independent replicates for each condition (n=3).

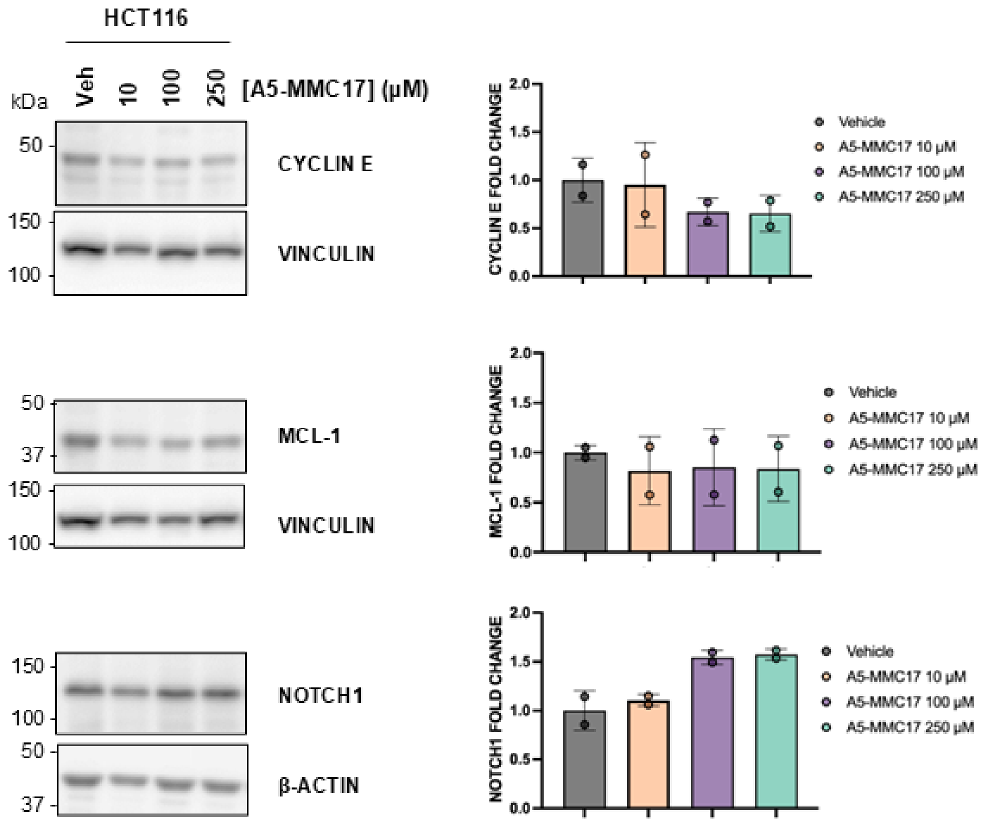

SUPPORTING DATA

**Supporting Data S1: Intact MS spectra of PhotoXplorer fragments vs FBW7 for phabits PhP007, PhP019, PhP006, PhP013, PhP093 and PhP060.**	Page 23
**Supporting Data S2: Intact MS spectra of PhotoXplorer fragments vs FBW7 for phabits PhP007, PhP019, PhP006, PhP013, PhP093 and PhP060.**	Page 26
**Supporting Data S3: SPR sensorgrams and dose-response fitted curves of the positive binders of FBW7.**	Page 29
**Supporting Data S4: HPLC of A5-MMC17 compound.**	Page 33
**Supporting Data S5: Detailed list of antibodies used for immunoblotting experiments.**	Page 35
**Supporting Data S6: Matrix-assisted Laser Desorption/Ionization (MALDI) of FBW7 and SDS polyacrylamide gel electrophoresis**	Page 36

Supporting Data S1. **Intact MS spectra of PhotoXplorer fragments vs FBW7 for Phabits PhP007, PhP019, PhP006, PhP013, PhP093 and PhP060.**

Intact protein masses were recorded by LC-MS using the Agilent 1100 series liquid chromatography and sample handling system. The chromatography was carried out on a Phenomenex SecurityGuard Widepore C4 4x3 mm cartridge. The eluents were: A, Water + 0.1 V/V% FA; B, ACN + 0.1 V/V% FA. The flow rate was set to 0.5 mL/min. The linear gradient started at 10% B; in 2.0 min it goes up to 65% B, flow rate also goes up to 1.0 mL/min; isocratic hold for 0.5min, same flow rate; it goes back to 10% B and 0.5 mL/min in 0.1 min; and it is kept for 1.4 min. Total time is 4.0 min. The eluent was injected into a Sciex x500b QTOF mass spectrometer (Sciex, MA, USA) system with electrospray ionization, positive mode. The source parameters are: curtain gas, 40 psi; ion source gas 1, 40 psi; ion source gas 2, 40 psi; temperature, 450 °C; ionspray voltage, 5500 V; declustering potential, 50 V. Other parameters are: time of flight (TOF) scan range, 500-2000 Da; accumulation time, 0.25 s; CAD gas, 7 arbitrary unit (AU); collision energy, 10 V.

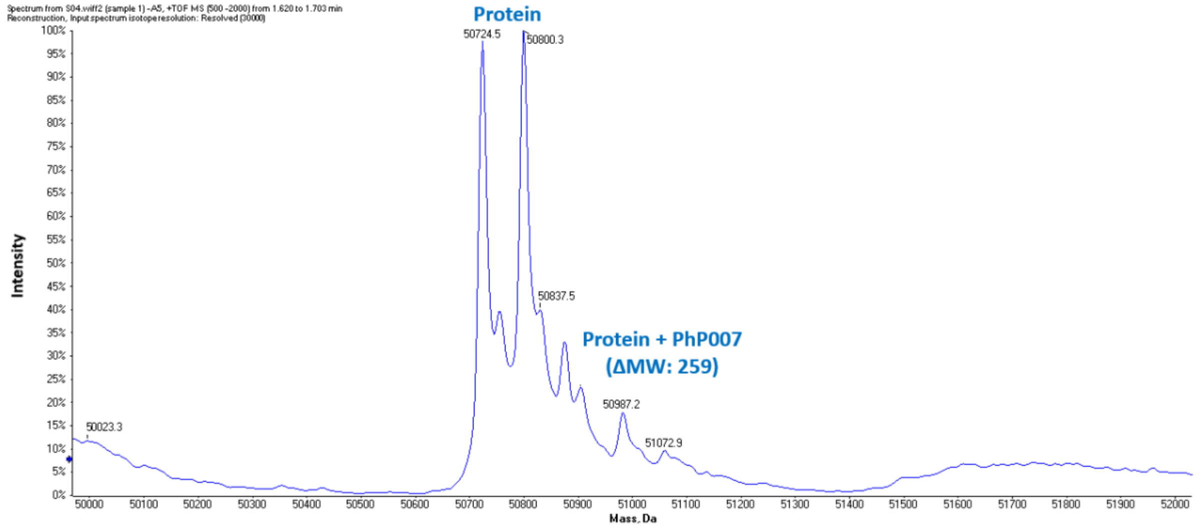
Protein
50724.5
50800.3
50837.5
Protein + PhP007
(ΔMW: 259)
50987.2
51072.9
50023.3
Intensity
Mass, Da

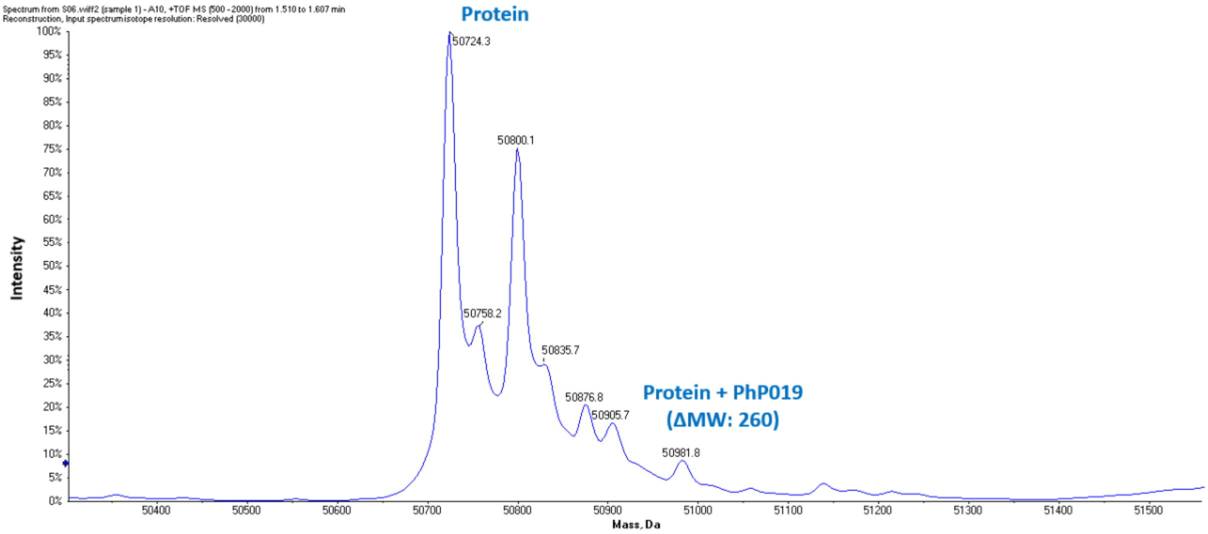
Protein
50724.3
50800.1
50758.2
50835.7
50876.8
50905.7
Protein + PhP019
(ΔMW: 260)
50981.8
Intensity
Mass, Da

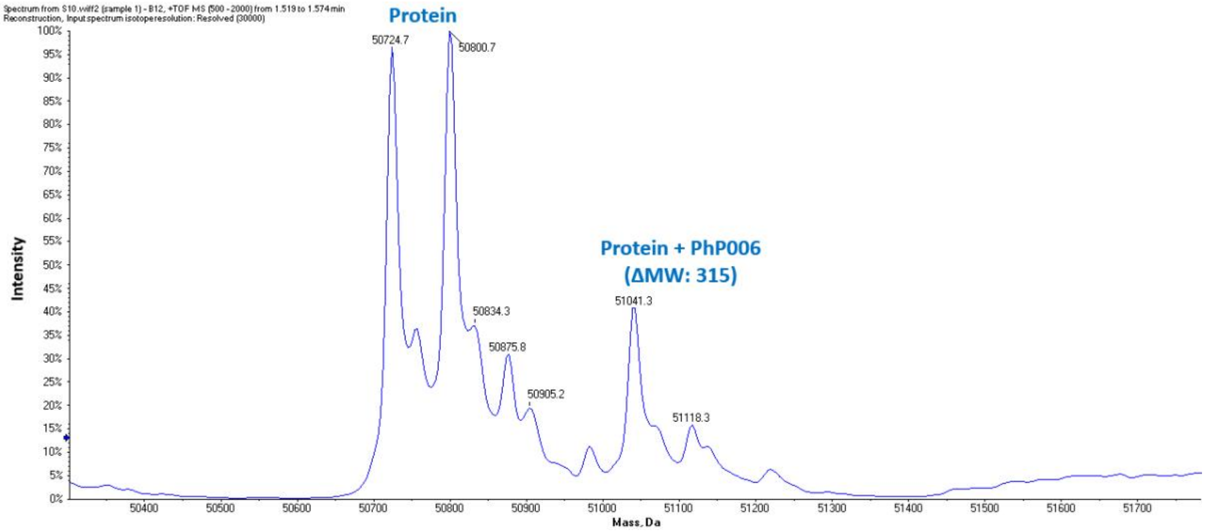
Protein
50724.7
50800.7
50834.3
50875.8
50905.2
Protein + PhP006
(ΔMW: 315)
51041.3
51118.3
Intensity
Mass, Da

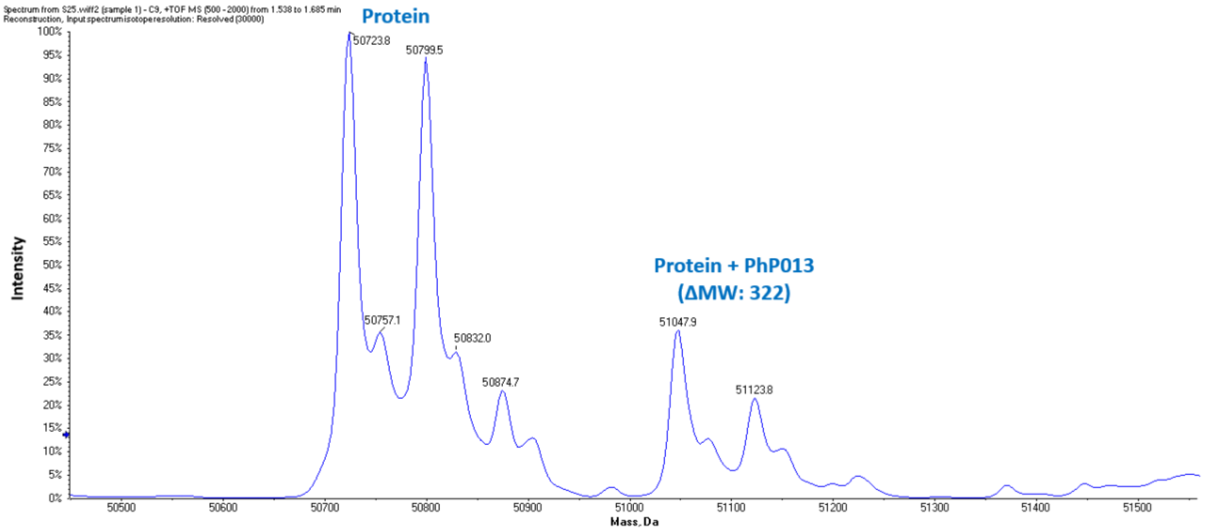
Protein
Protein + PhP013
(ΔMW: 322)
50723.8
50799.5
50757.1
50832.0
50874.7
51047.9
51123.8
Intensity
Mass, Da

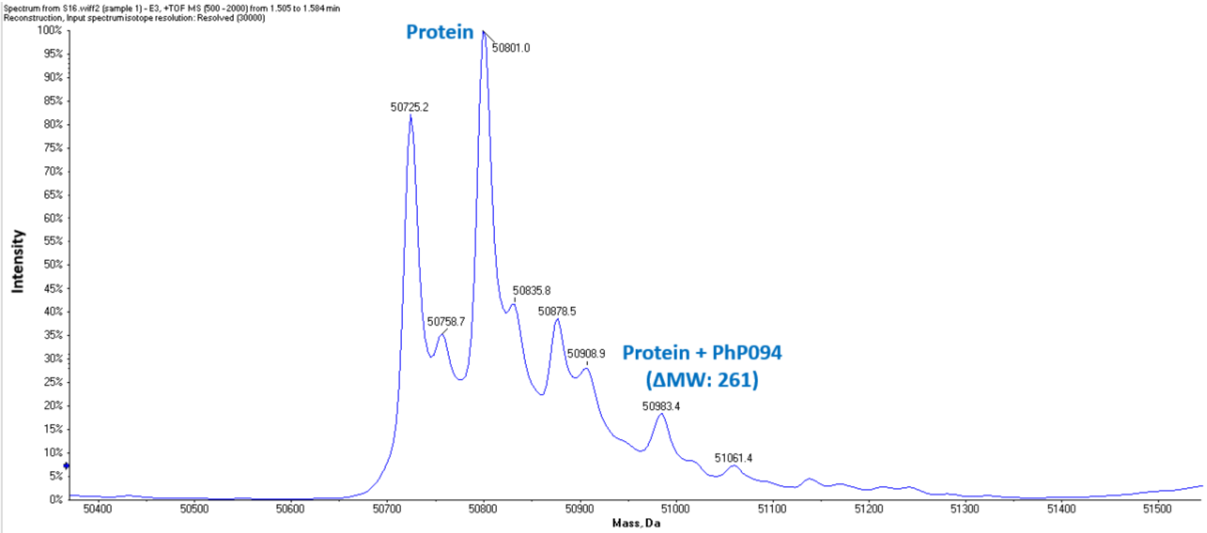
Protein
Protein + PhP094
(ΔMW: 261)
50801.0
50725.2
50835.8
50878.5
50758.7
50908.9
50983.4
51061.4
Intensity
Mass, Da

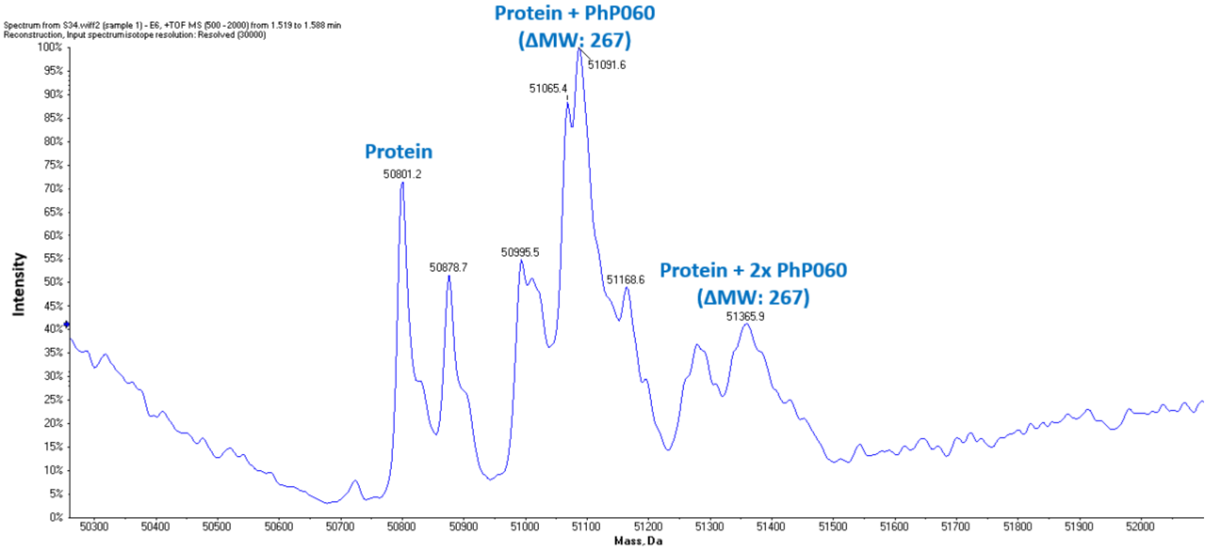
Protein + PhP060
(ΔMW: 267)
Protein
Protein + 2x PhP060
(ΔMW: 267)
51091.6
51065.4
50801.2
50995.5
50878.7
51168.6
51365.9
Intensity
Mass, Da

Supporting Data S2. **MS spectra of digested FBW7 after labelling by PhotoXplorer hit fragments.**

Samples were analysed by a Triple TOF 5600+ hybrid Quadrupole-TOF LC-MS/MS system (Sciex, MA, USA) equipped with a DuoSpray IonSource coupled with a Shimadzu Prominence LC20 UFLC (Shimadzu, Japan) system consisting of quaternary pump, an autosampler and a thermostated column compartment. Data acquisition and processing were performed using Analyst TF software version 1.7.1 (AB Sciex Instruments, CA, USA). Chromatographic separation was achieved on the Discovery® BIO Wide Pore C-18-5 (250 mm × 2.1 mm, 5 µm, 300 Å) HPLC column. Sample was eluted in gradient elution mode using solvent A (0.1% formic acid in water) and solvent B (0.1% formic acid in ACN). The initial condition was 5% B for 7 min, followed by a linear gradient to 90% B by 48 min, from 55 to 63 min 90% B was retained; and from 63 to 65 min back to initial condition with 5 % eluent B and retained for 10 min. Flow rate was set to 0.2 ml/min. The column temperature was 40 °C and the injection volume was 15 µL. Nitrogen was used as the nebulizer gas (GS1), heater gas (GS2), and curtain gas with the optimum values set at 35, 35 and 35 (arbitrary units), respectively. The source temperature was 350 °C and the spray voltage was set to 5000 V. Advanced Information Dependent Acquisition (IDA) mode was used on the TripleTOF 5600+ system to obtain MS/MS spectra on the 8 most abundant parent ions present in the TOF survey scan. In IDA LC-MS/MS experiment the mass spectra and tandem mass spectra were recorded in “high-sensitivity” mode with a resolution of ~35,000 full-width half-maximum. In first period (positive TOF MS mode) the data were acquired in the mass range of m/z=300 to 2500, with 0.1 s accumulation time. Declustering potential value was set to 60 V. The intensity threshold for precursor ion selection in TOF survey scan mode was 1000 cps. In MS2 experiment (Product Ion scan mode): the mass range was m/z=50 to 3000, with an accumulation time of 0.1 sec. PeakView® V.2.2 software (version 2.2, Sciex) and Biologics Explorer software (version 750 3.0.3, Sciex) were used to assign and evaluate the peaks in the MSMS spectra.

MS/MS of the peptide DLLQAAQTCR modified by PhP006, at Fbw7Q306 (44Q) or Fbw7C308 (46C).				
**Peptide**	**Start**	**End**	**RT (min)**	**Calc. peptide mass (Da)**
DLLQAAQTCR	38	47	29.93	1433.6557

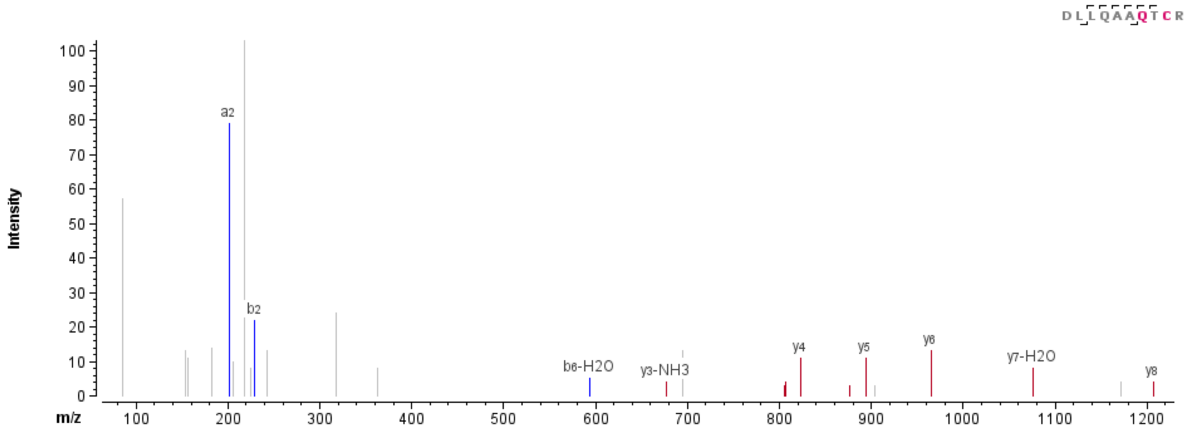

MS/MS of the peptide GHDDHVITCLQFCGNR modified by PhP013, at Fbw7C390 (128C)				
**Peptide**	**Start**	**End**	**RT (min)**	**Calc. peptide mass (Da)**
GHDDHVITCLQF CGNR	116	131	32.18	2193.985

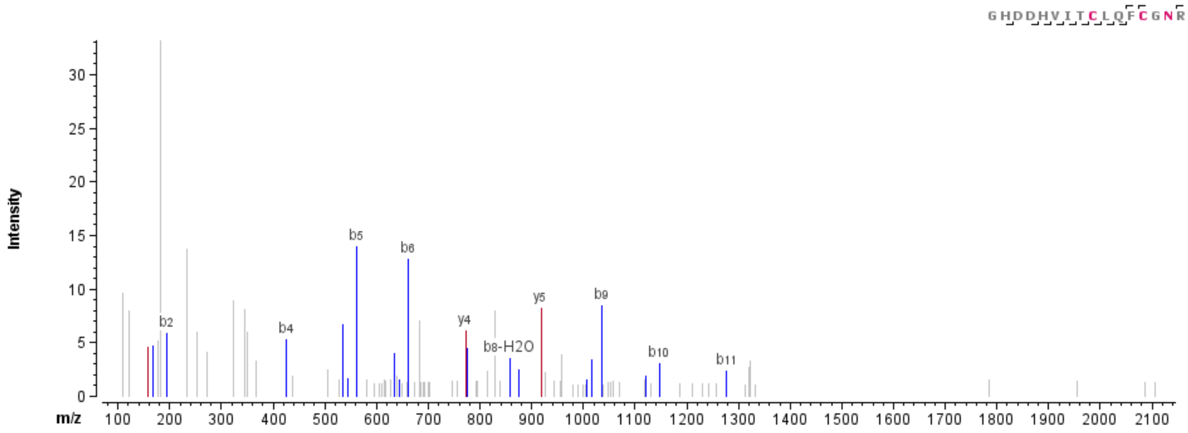

MS/MS of the peptide DLLQAAQTCR modified by PhP060, at: Fbw7T307 (45T) or Fbw7C308 (46C) or Fbw7R309 (47R) and MS/MS of the peptide TGQCLQTLQGPNK modified by PhP060, at Fbw7C613 (351C).				
**Peptide**	**Start**	**End**	**RT (min)**	**Calc. peptide mass (Da)**
DLLQAAQTCR	38	47	30.19	1386.6364
TGQCLQTLQGP NK	348	360	23.66	1655.7739

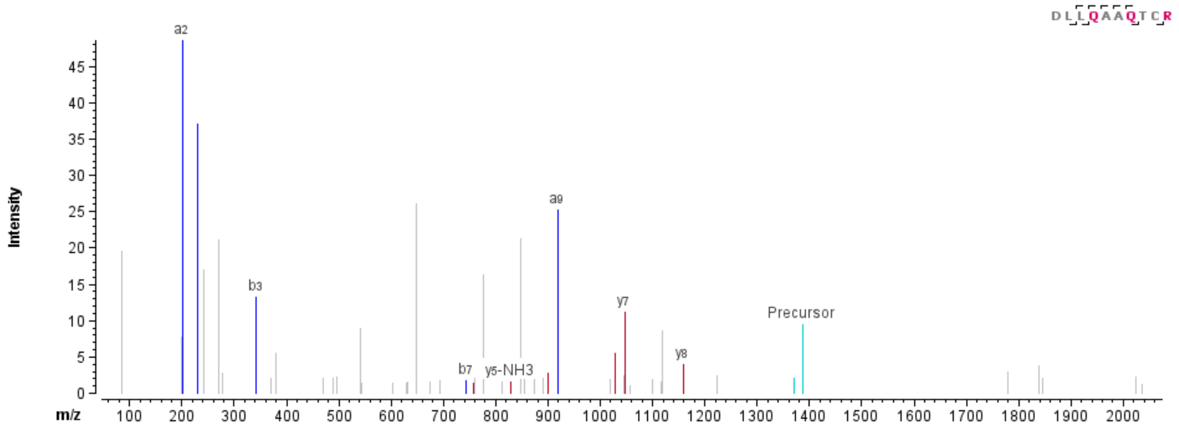
DLLQAAQTCR
Intensity
m/z
a2
b3
b7
y5-NH3
a9
y7
y8
Precursor

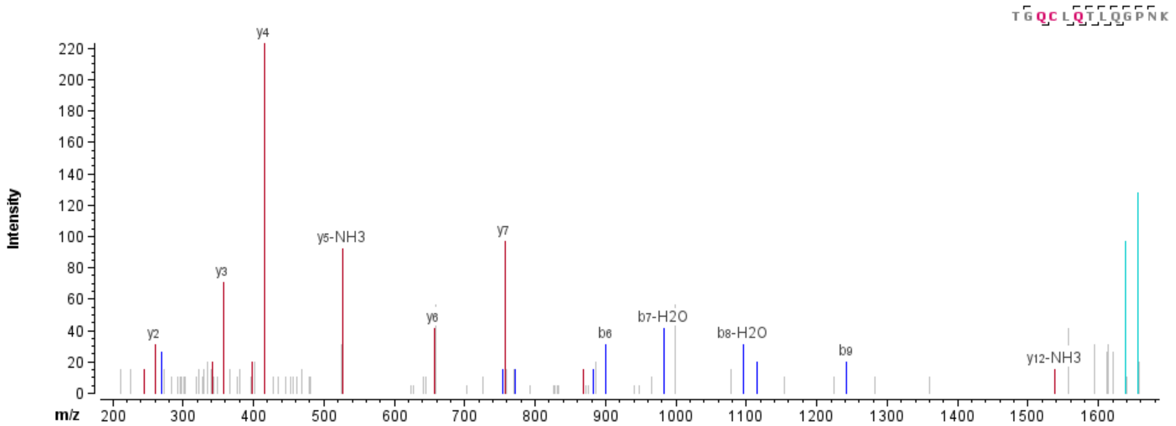
TGQCLQTLQGPNK
Intensity
m/z
y2
y3
y4
y5-NH3
y6
y7
b6
b7-H2O
b8-H2O
b9
y12-NH3

Supporting Data S3. **SPR sensorgrams and dose-response fitted curves of the positive binders of FBW7.**

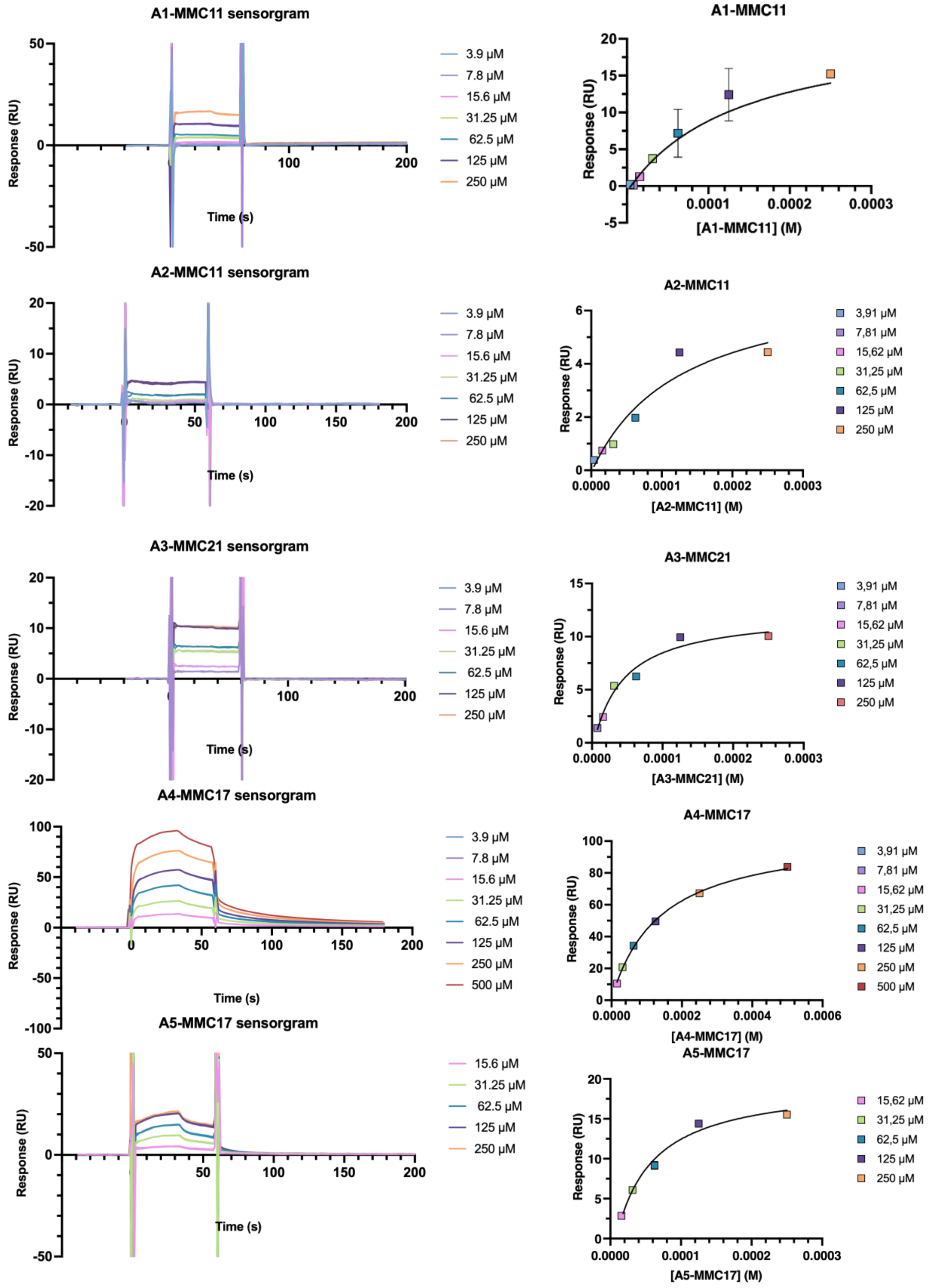

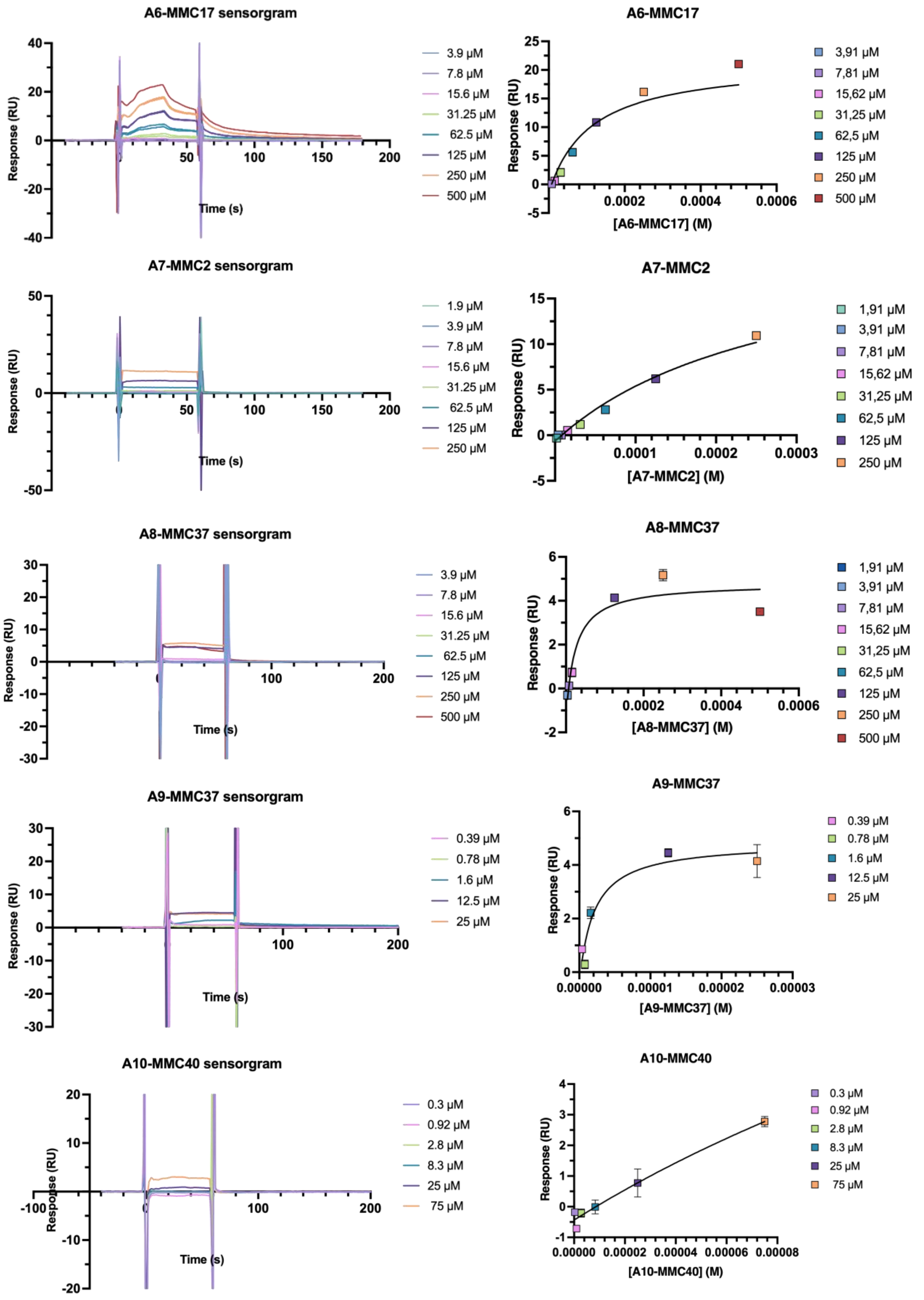
A6-MMC17 sensorgram
Response (RU)
Time (s)
3.9 µM
7.8 µM
15.6 µM
31.25 µM
62.5 µM
125 µM
250 µM
500 µM
A6-MMC17
Response (RU)
[A6-MMC17] (M)
3,91 µM
7,81 µM
15,62 µM
31,25 µM
62,5 µM
125 µM
250 µM
500 µM
A7-MMC2 sensorgram
Response (RU)
Time (s)
1.9 µM
3.9 µM
7.8 µM
15.6 µM
31.25 µM
62.5 µM
125 µM
250 µM
A7-MMC2
Response (RU)
[A7-MMC2] (M)
1,91 µM
3,91 µM
7,81 µM
15,62 µM
31,25 µM
62,5 µM
125 µM
250 µM
A8-MMC37 sensorgram
Response (RU)
Time (s)
3.9 µM
7.8 µM
15.6 µM
31.25 µM
62.5 µM
125 µM
250 µM
500 µM
A8-MMC37
Response (RU)
[A8-MMC37] (M)
1,91 µM
3,91 µM
7,81 µM
15,62 µM
31,25 µM
62,5 µM
125 µM
250 µM
500 µM
A9-MMC37 sensorgram
Response (RU)
Time (s)
0.39 µM
0.78 µM
1.6 µM
12.5 µM
25 µM
A9-MMC37
Response (RU)
[A9-MMC37] (M)
0.39 µM
0.78 µM
1.6 µM
12.5 µM
25 µM
A10-MMC40 sensorgram
Response (RU)
Time (s)
0.3 µM
0.92 µM
2.8 µM
8.3 µM
25 µM
75 µM
A10-MMC40
Response (RU)
[A10-MMC40] (M)
0.3 µM
0.92 µM
2.8 µM
8.3 µM
25 µM
75 µM

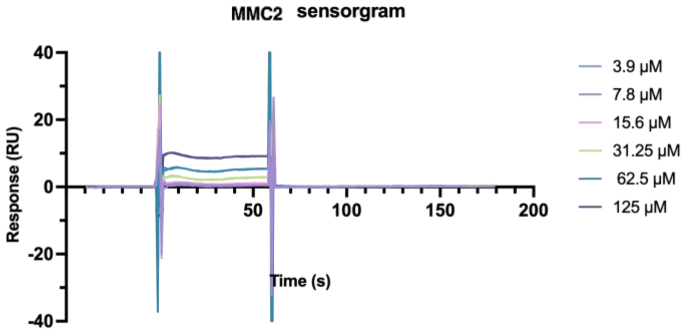
MMC2 sensorgram
Response (RU)
Time (s)
40
20
0
-20
-40
50
100
150
200
3.9 µM
7.8 µM
15.6 µM
31.25 µM
62.5 µM
125 µM

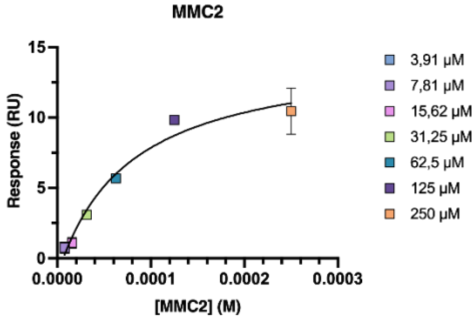
MMC2
Response (RU)
[MMC2] (M)
15
10
5
0
0.0000
0.0001
0.0002
0.0003
3,91 µM
7,81 µM
15,62 µM
31,25 µM
62,5 µM
125 µM
250 µM

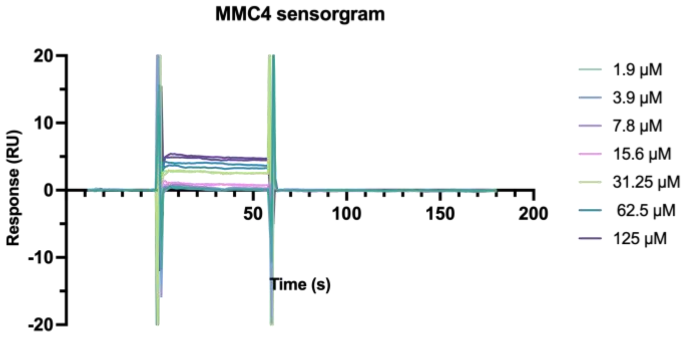
MMC4 sensorgram
Response (RU)
Time (s)
20
10
0
-10
-20
50
100
150
200
1.9 µM
3.9 µM
7.8 µM
15.6 µM
31.25 µM
62.5 µM
125 µM

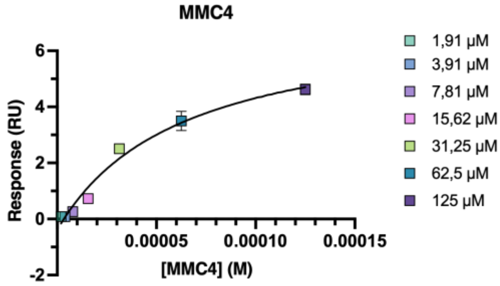
MMC4
Response (RU)
[MMC4] (M)
6
4
2
0
-2
0.00005
0.00010
0.00015
1,91 µM
3,91 µM
7,81 µM
15,62 µM
31,25 µM
62,5 µM
125 µM

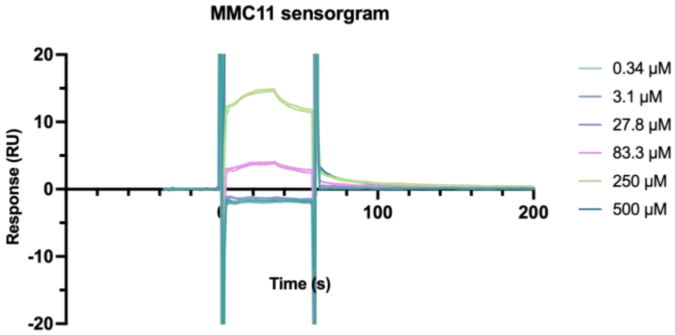
MMC11 sensorgram
Response (RU)
Time (s)
20
10
0
-10
-20
100
200
0.34 µM
3.1 µM
27.8 µM
83.3 µM
250 µM
500 µM

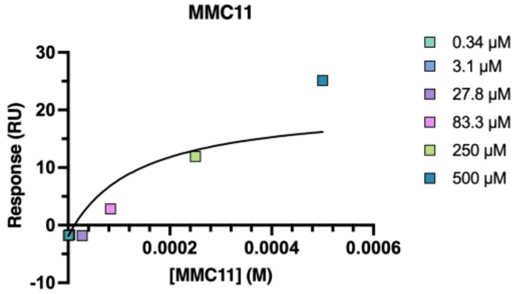
MMC11
Response (RU)
[MMC11] (M)
30
20
10
0
-10
0.0002
0.0004
0.0006
0.34 µM
3.1 µM
27.8 µM
83.3 µM
250 µM
500 µM

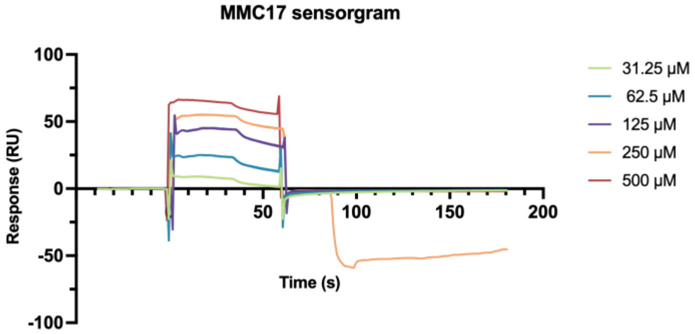
MMC17 sensorgram
Response (RU)
Time (s)
100
50
0
-50
-100
50
100
150
200
31.25 µM
62.5 µM
125 µM
250 µM
500 µM

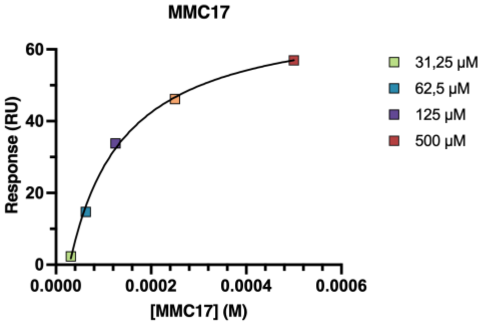
MMC17
Response (RU)
[MMC17] (M)
60
40
20
0
0.0000
0.0002
0.0004
0.0006
31,25 µM
62,5 µM
125 µM
500 µM

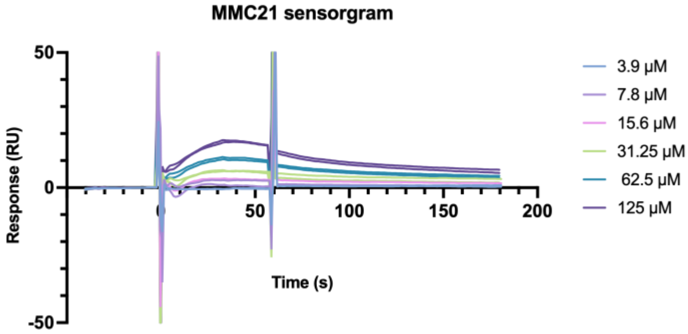
MMC21 sensorgram
Response (RU)
Time (s)
50
0
-50
50
100
150
200
3.9 µM
7.8 µM
15.6 µM
31.25 µM
62.5 µM
125 µM

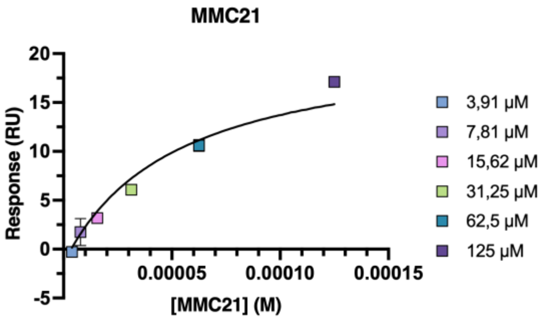
MMC21
Response (RU)
[MMC21] (M)
20
15
10
5
0
-5
0.00005
0.00010
0.00015
3,91 µM
7,81 µM
15,62 µM
31,25 µM
62,5 µM
125 µM

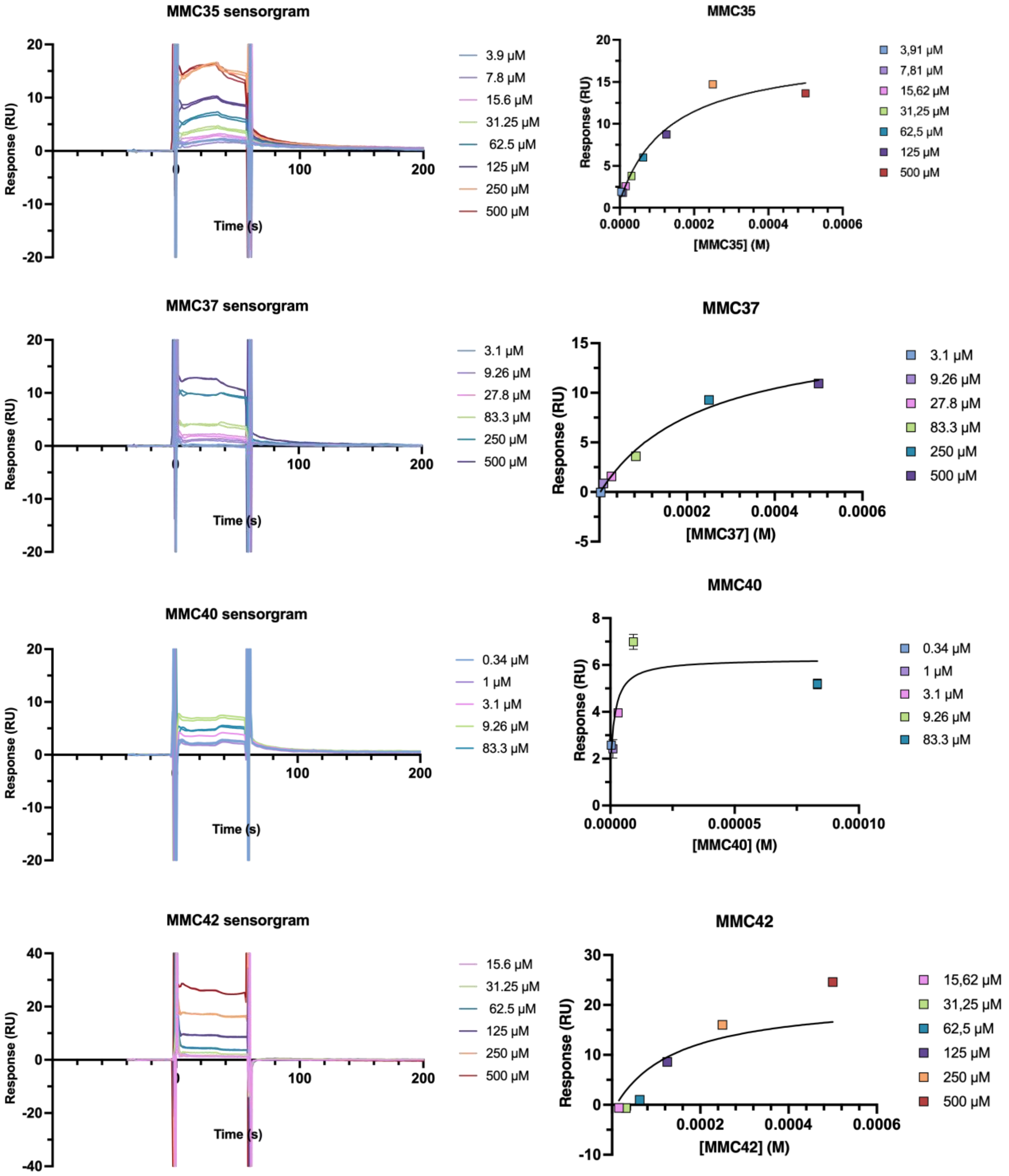
MMC35 sensorgram
Response (RU)
Time (s)
3.9 µM
7.8 µM
15.6 µM
31.25 µM
62.5 µM
125 µM
250 µM
500 µM
MMC35
[MMC35] (M)
3,91 µM
7,81 µM
15,62 µM
31,25 µM
62,5 µM
125 µM
500 µM
MMC37 sensorgram
3.1 µM
9.26 µM
27.8 µM
83.3 µM
250 µM
500 µM
MMC37
[MMC37] (M)
MMC40 sensorgram
0.34 µM
1 µM
3.1 µM
9.26 µM
83.3 µM
MMC40
[MMC40] (M)
MMC42 sensorgram
15.6 µM
31.25 µM
62.5 µM
125 µM
250 µM
500 µM
MMC42
[MMC42] (M)
15,62 µM
31,25 µM
62,5 µM
125 µM
250 µM
500 µM

Supporting Data S4. **HPLC-MS and 1H-RMN of A5-MMC17 compound.**

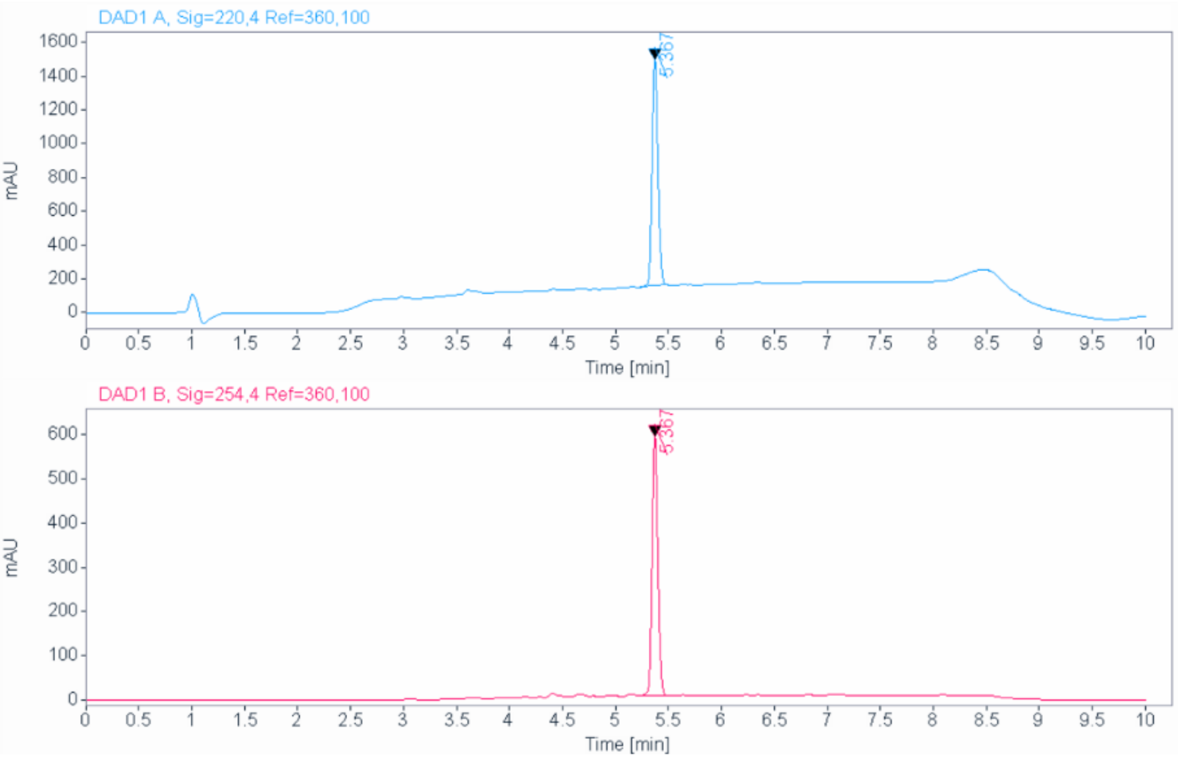

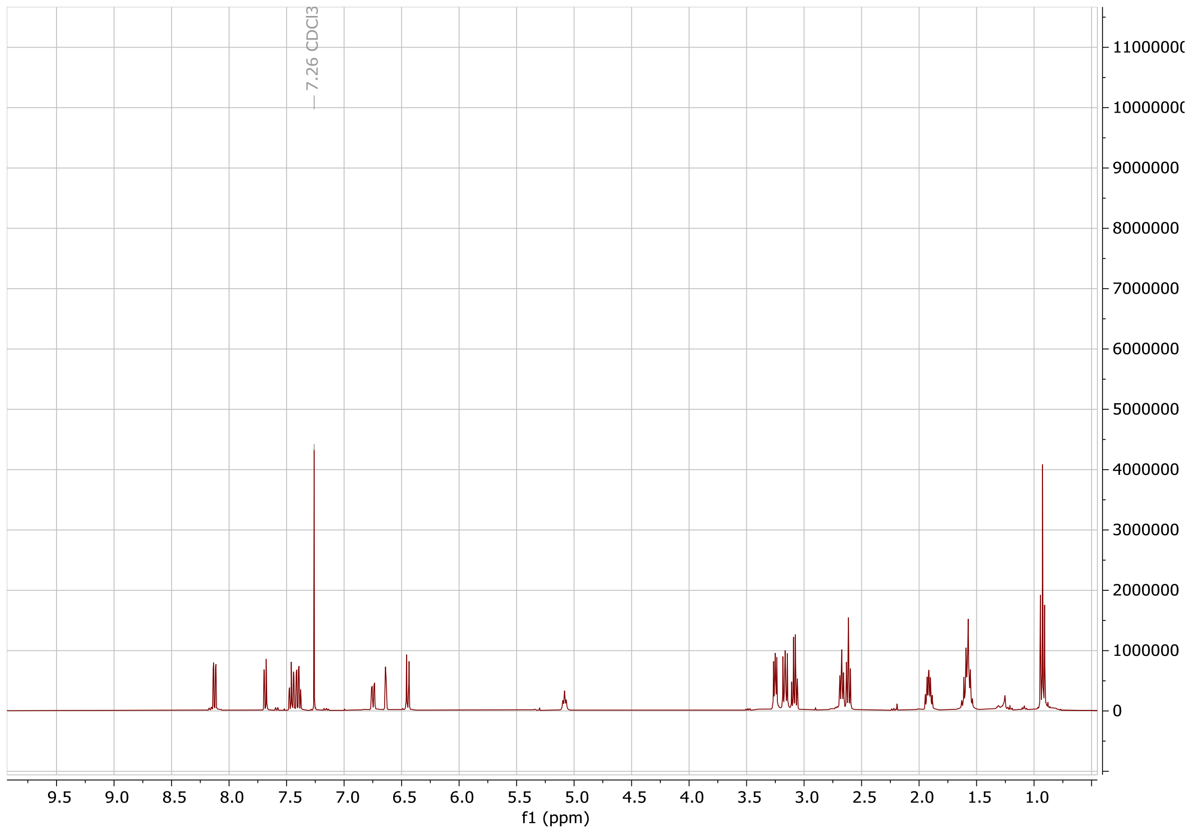

^{1}H NMR (400 MHz, $CDCl_3$) δ (ppm): 8.12 (dd, *J* = 7.6 Hz, *J'* = 1.6 Hz , 1H), 7.68 (dd, *J* = 7.6 Hz, *J'* = 1.2 Hz, 1H), 7.45 (ddd, *J* = *J'* = 7.6 Hz, *J"* = 1.2 Hz, 1H), 7.40 (ddd, *J* = *J'* = 7.6 Hz, *J''* = 1.6 Hz, 1H), 6.74 (dd, *J* = 8.4 Hz, *J'* = 2.0 Hz, 1H), 6.63 (d, *J* =2 Hz, 1H), 6.44 (d, *J* = 8.4 Hz, 1H), 5.09 (t, *J* = 6 Hz, 1H), 3.19 (t, *J* = 5.6 Hz, 2H), 3.16 (t, *J* = 7.6 Hz, 2H), 3.08 (dd, *J* = *J'* = 7.2 Hz, 2H), 2.67 (t, *J* = 6.4 Hz, 2H), 2.61 (t, *J* = 6.8 Hz, 2H), 1.91 (tt, *J* = *J'* = 6.4 Hz, 2H), 1.58 (tq, *J* = *J'* = 7.6 Hz, 2H), 0.92 (t, *J* = 7.2 Hz, 3H).

Supporting Data S5. **Detailed list of antibodies used for immunoblotting experiments.**

Antibody	Reference	Dilution	Incubation
anti-c-Myc	Abcam (#ab32072)	1:1000 in TBS-T	o/n, 4ºC
anti-Cyclin E	Abcam (#ab33911)	1:1000 in TBS-T + 5% non-fat dry milk	o/n, 4ºC
anti-MCL-1	Cell Signalling Technology (#5453)	1:1000 in TBS-T + 5% non-fat dry milk	o/n, 4ºC
anti-Notch1	Abcam (#ab52627)	1:1000 TBS-T + 5% non-fat dry milk	o/n, 4ºC
Anti-c-Jun	Cell Signalling Technology (#9165)	1:1000 in TBS-T + 5% non-fat dry milk	o/n, 4ºC
anti-Vinculin	Abcam (#ab129002)	1:1000 in TBS-T	o/n, 4ºC
anti-β-actin	Abcam (#ab115777)	1:1000 in TBS-T	o/n, 4ºC
Goat Anti-Rabbit IgG (H+L), HRP	Jackson Immunoresearch (#111-035-003)	c-Myc, Vinculin, β-actin, (1:10000); MCL-1, Notch1, c-Jun (1:5000); Cyclin E (1:2000)	2h, RT

***Supporting Data S6*: Matrix-assisted Laser Desorption/Ionization (MALDI) of FBW7 and SDS polyacrylamide gel electrophoresis.**

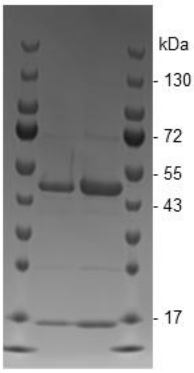

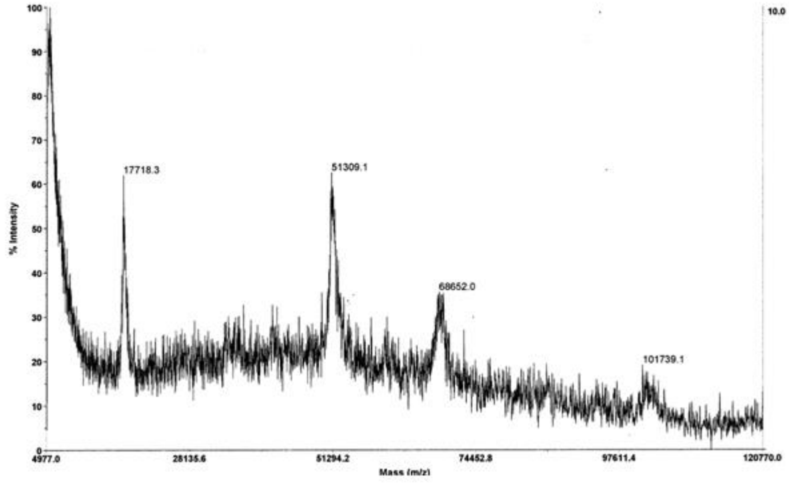

Supplement: Supplementary file 1 — Supporting Information [file ADVS-12-e06068-s001.pdf]
